# *Streptococcus pneumoniae* serotypes that frequently colonise the human nasopharynx are common recipients of penicillin-binding protein gene fragments from *Streptococcus mitis*

**DOI:** 10.1099/mgen.0.000622

**Published:** 2021-09-22

**Authors:** Akuzike Kalizang'oma, Chrispin Chaguza, Andrea Gori, Charlotte Davison, Sandra Beleza, Martin Antonio, Bernard Beall, David Goldblatt, Brenda Kwambana-Adams, Stephen D. Bentley, Robert S. Heyderman

**Affiliations:** ^1^​ NIHR Global Health Research Unit on Mucosal Pathogens, Division of Infection & Immunity, University College London, London, UK; ^2^​ Parasites and Microbes, Wellcome Sanger Institute, Hinxton, UK; ^3^​ Darwin College, University of Cambridge, Silver Street, Cambridge, UK; ^4^​ Department of Clinical Infection, Microbiology and Immunology, Institute of Infection, Veterinary and Ecological Sciences, University of Liverpool, Liverpool, UK; ^5^​ Department of Genetics and Genome Biology, University of Leicester, Leicester, UK; ^6^​ Medical Research Council Unit The Gambia at London School of Hygiene & Tropical Medicine, World Health Organization, Collaborating Centre for New Vaccines Surveillance, Banjul, Gambia; ^7^​ Centers for Disease Control and Prevention, National Center for Immunization and Respiratory Diseases, Division of Bacterial Diseases, Atlanta, GA, USA; ^8^​ University College London, Great Ormond Street Institute of Child Health, London, UK

**Keywords:** beta-lactam resistance, horizontal gene transfer, penicillin binding protein genes, *Streptococcus mitis*, *Streptococcus oralis*, *Streptococcus pneumoniae*

## Abstract

*
Streptococcus pneumoniae* is an important global pathogen that causes bacterial pneumonia, sepsis and meningitis. Beta-lactam antibiotics are the first-line treatment for pneumococcal disease, however, their effectiveness is hampered by beta-lactam resistance facilitated by horizontal genetic transfer (HGT) with closely related species. Although interspecies HGT is known to occur among the species of the genus *Streptococcus *, the rates and effects of HGT between *Streptococcus pneumoniae* and its close relatives involving the penicillin binding protein (*pbp*) genes remain poorly understood. Here we applied the fastGEAR tool to investigate interspecies HGT in *pbp* genes using a global collection of whole-genome sequences of *Streptococcus mitis *, *Streptococcus oralis* and *S. pneumoniae *. With these data, we established that pneumococcal serotypes 6A, 13, 14, 16F, 19A, 19F, 23F and 35B were the highest-ranking serotypes with acquired *pbp* fragments. *S. mitis* was a more frequent pneumococcal donor of *pbp* fragments and a source of higher *pbp* nucleotide diversity when compared with *S. oralis *. Pneumococci that acquired *pbp* fragments were associated with a higher minimum inhibitory concentration (MIC) for penicillin compared with pneumococci without acquired fragments. Together these data indicate that *S. mitis* contributes to reduced β-lactam susceptibility among commonly carried pneumococcal serotypes that are associated with long carriage duration and high recombination frequencies. As pneumococcal vaccine programmes mature, placing increasing pressure on the pneumococcal population structure, it will be important to monitor the influence of antimicrobial resistance HGT from commensal streptococci such as *S. mitis *.

## Data Summary

*Streptococcus pneumoniae* genome sequences used in this project are listed in File S1 available in the online version of this article and can be accessed under BioProjects PRJEB2417, PRJEB2632, PRJEB3084 and PRJEB2357. Accession numbers for all publicly available *S. mitis* and *S. oralis* genome sequences are also listed in File S1 and additional genome sequences used in this project can be accessed under BioProjects PRJNA480039, PRJEB42564 and PRJEB42963.

**Impact Statement**

*Streptococcus pneumoniae,* also known as the pneumococcus, is a global pathogen that is regarded as a serious antimicrobial resistance threat. The transfer of genetic material from *Streptococcus mitis* to the pneumococcus at genes that confer resistance to penicillin has contributed to pneumococcal antimicrobial resistance. However, the effect of genetic transfer between *S. mitis* and different *S. pneumoniae* serotypes at the penicillin genes is uncertain. We applied genetic transfer inference software to investigate genetic transfer within the penicillin genes using 1000 strains of *S *. *pneumoniae* from global carriage studies, and a comprehensive dataset of publicly available *S. mitis* and *S. oralis* whole genome sequences. We identified recent genetic transfer at the penicillin genes mostly from *S. mitis* to *S. pneumoniae,* and *S. pneumoniae* serotypes associated with long carriage duration in the human upper respiratory tract were the main recipients. *S. pneumoniae* strains with acquired genetic material were also associated with reduced susceptibility to penicillin. The findings will serve as a foundation for monitoring genetic transfer of antimicrobial resistance in the pneumococcus, particularly in regions where the pneumococcus is under increasing pressure from antibiotic use and pneumococcal conjugate vaccine uptake.

## Introduction

*Streptococcus pneumoniae* is a common nasopharyngeal commensal particularly among children and HIV-affected adult populations in sub-Saharan Africa (sSA) [1, 2]. Globally, the pathogen is estimated to be responsible for over 318 000 deaths annually among children less than 5 years of age due to bacteraemic pneumonia, sepsis and meningitis [1, 3]. Pneumococcal serotypes associated with invasive pneumococcal disease (IPD) and antibiotic resistance have been targeted by pneumococcal conjugate vaccines (PCVs) [4, 5] and their rollout has led to a reduction of vaccine serotype (VT) IPD and pneumococcal antimicrobial resistance [6, 7]. However, the pneumococcus has been able to escape these interventions, leading to the expansion of resistant non-vaccine serotypes (NVTs) [8, 9], residual carriage of VTs [10, 11] and serotype switching [12–15].

Antibiotic resistant pneumococcal lineages that continue to evade vaccine interventions are of considerable public health concern [16]. In the United States the post-PCV introduction era has seen the expansion of serotype 35B sequence type (ST) 558 [17], a resistant NVT lineage that has been associated with IPD in the region, as well as a 35B switch variant that has recently emerged [8, 17, 18]. In high disease burden regions, immunised children continue to carry antibiotic resistant VTs, which include serotypes 19A and 19F, despite good vaccine coverage [11, 19–21]. Additionally, multidrug-resistant (MDR) *S. pneumoniae* clones defined by the Pneumococcal Molecular Epidemiology Network (PMEN) [22], such as Sweden^15A^-25/ST63 and Netherlands^15B^-37/ST199, spreading globally continue to remain important causes of invasive disease in the post-PCV13 era [23–25].

Beta-lactam (β-lactam) antibiotics, including penicillin, are the first line treatment for pneumococcal disease, however, the emergence of β-lactam resistance presents a global threat [26]. The mechanism of pneumococcal β-lactam resistance involves alterations in the transpeptidase domains (TPDs) located within the penicillin-binding protein (*pbp*) genes, namely *pbp1a*, *pbp2b* and *pbp2x* [27–29]. Results from several genomic studies have indicated that the *pbp* genes (*pbp1a, pbp2b, pbp2x*) within the pneumococcal genome are hotspots for intraspecies recombination events [13, 30]. Nucleotide sequencing studies in the early 1990s provided the first evidence of interspecies genetic transfer between the pneumococcus and *Streptococcus mitis* [31–43]. More recently, the application of methods for detecting horizontal genetic transfer (HGT) [44, 45] have provided further evidence for the emergence of β-lactam resistance in the pneumococcus facilitated by HGT with closely related commensal streptococci such as *S. mitis* and *Streptococcus oralis* [46–48]. These species are predominant in the oropharynx [49, 50] and are of less pathogenic potential than the pneumococcus, however, they are widely regarded as reservoirs for antibiotic resistance determinants for the pneumococcus.

Although interspecies HGT at *pbp* gene loci is known to occur among the species of the genus *Streptococcus* [31–42, 48], the rates and effects of HGT at *pbp* genes between closely related non-pneumococcal streptococci and individual pneumococcal carriage serotypes, genotypes and sequence types is uncertain. We propose the hypothesis that pneumococcal serotypes that frequently colonise the human nasopharynx for long durations and have higher proportions of observed recombination, are the predominant recipients of *pbp* gene fragments from commensal streptococci, resulting in reduced pneumococcal penicillin susceptibility.

To detail the evolution of pneumococcal β-lactam resistance among lineages that continue to evade vaccine intervention, we have therefore investigated HGT at *pbp*, housekeeping and other antimicrobial resistance (AMR) genes among *S. mitis, S. oralis* and pneumococcal strains obtained through pneumococcal disease surveillance programmes and carriage studies including the Global Pneumococcal Sequencing (GPS) project [51]. Our study highlights *S. mitis* as a more frequent donor of genetically diverse *pbp* gene fragments to *S. pneumoniae* compared with *S. oralis *. The horizontal acquisition of the *pbp* fragments mostly occurs among pneumococcal serotypes associated with prolonged nasopharyngeal carriage duration and is associated with reduced susceptibility to β-lactam antibiotics.

## Methods

### Bacterial genome selection

All publicly available *Streptococcus mitis* and *Streptococcus oralis* genome assemblies were downloaded from the NCBI database and were from both carriage and invasive disease. The source was unknown for some of the strains (See File S1 for a list of accession numbers and origins). Additional *S. mitis* (*n*=31) and *S. oralis* (*n*=23) genome assemblies from nasopharyngeal carriage were obtained from MRC The Gambia (Banjul, Gambia), and whole genome sequencing and species identification was done at the University of Leicester (File S1). *S. mitis* genome assemblies from University College London (*n*=4) and the US Centres for Disease Control and Prevention (CDC; *n*=5) obtained from carriage were also included in the analysis (File S1). In total we obtained 174 *S *. *mitis* and 135 *S *. *oralis* genomes for species confirmation. It should be noted that very few non-pneumococcal streptococci have been sequenced in comparison to the pneumococcus, therefore, the undersampled *S. mitis* and *S. oralis* datasets are a limitation in investigating the extent of interspecies HGT.

To obtain adequate coverage of global pneumococcal carriage serotypes, pneumococcal carriage datasets from the Global Pneumococcal Sequencing (GPS) Project (http://www.pneumogen.net/gps/) were used to sample *S. pneumoniae* genome assemblies (*n*=1000) (File S1). Pneumococcal carriage datasets from Malawi, Gambia, Thailand, the UK and the USA were selected to obtain representation across different continents, and 200 genomes were randomly selected from each dataset.

### Analysis platforms and genomic analysis

Internal automated pipelines and computing clusters developed and maintained by the Wellcome Sanger Institute (WSI; Hinxton, Cambridgeshire, UK) were used for whole genome sequence analysis, where the genomic data was all analysed at the same time [51, 52]. Additional genomic analyses were carried out at UCL and through the genomic surveillance PathogenWatch application (https://pathogen.watch/).

Taxonomic classification of *S. pneumoniae *, *S. mitis* and *S. oralis* genome assemblies was done using KRAKEN v1.0 [53] to confirm species identity. Genome quality was determined using the quality assessment tool for genome assemblies (QUAST v5.0.2) [54]. Genomes that were not assigned as *S. pneumoniae *, *S. mitis* and *S. oralis* were excluded from the analysis. The streptococcal genome assemblies were then annotated using Prokka v1.13.4 [55] as part of the WSI annotation pipeline, and pangenome analysis was conducted using Roary v3.13.0 [56] in the WSI pangenome pipeline to obtain core genome alignments. Pangenome analysis was run for the three streptococcal species together and separately for the pneumococcal strains to obtain two core genome alignments. Capsular serotyping and sequence typing based on the pneumococcal multilocus sequence typing scheme (MLST) were conducted using SeroBA v1.0.1 [57] and BIGsdb [58] respectively, while determination of newly proposed nomenclature by the GPS project of Global Pneumococcal Sequence Clusters (GPSCs) for internationally distributed pneumococcal lineages [59] was done using the PathogenWatch application (https://pathogen.watch/).

### Phylogenetic analysis

Multiple sequence alignments of polymorphic nucleotide sites were generated from the core genome alignments inferred by Roary pangenome analysis pipeline using snp-sites v2.5.1 [60]. The alignment of polymorphic sites was then used to reconstruct maximum-likelihood phylogenies using fasttree v2.1.10 [61]. We used the generalized time-reversible model of nucleotide evolution to generate the phylogenies, which were visualized and annotated using the online Interactive Tree of Life (iToL) software v3.0 [62] and microreact v5.93.0 [63]. Nucleotide-blast v2.10.1 [64] was used to extract *pbp1a*, *pbp2b* and *pbp2x* gene sequences. The *pbp* sequences were then aligned using muscle v3.8.1551 within the Molecular Evolutionary Genetics Analysis (mega) software v10.0 [65]. Population clustering of the *pbp* gene sequences was inferred using Bayesian Analysis of Population Structure (BAPS) [66] implemented via the fastGEAR tool [67]. Phylogenetic trees based on the *pbp* gene alignments were similarly reconstructed and visualised using the methods and parameters described above.

### Nucleotide sequence polymorphism and *pbp* binding motif analysis

The analysis of polymorphisms in *pbp1a*, *pbp2b* and *pbp2x* nucleotide sequences was conducted for *S. pneumoniae *, *S. mitis* and *S. oralis* using DnaSP v6 [68] to estimate the number of segregating sites (S), nucleotide diversity (π) and average number of pair-wise nucleotide differences within a population (K). We used a sliding window of 100 bases with a step size of 25 bp to calculate the nucleotide diversity of *pbp* genes from each species, and the respective plots were generated using the package ggplot2 [69] in R v3.6.3 [70]. Amino acid residues in the TPDs of *pbp1a*, *pbp2b* and *pbp2x* active binding motifs were analysed for each species using mega v10.0 and compared with the motifs of the β-lactam susceptible *S. pneumoniae* R6 (GenBank Accession GCA000007045.1). The domain regions used for the analysis were ^370^STMK^373^, ^428^SRNVP^432^, ^574^KTG^576^ for *pbp*1a, ^391^SVVK^394^, ^448^SSNT^451^ and ^620^KTGTA^624^ for *pbp*2b and ^337^STMK^340^, ^393^AHSSNV^398^ and ^546^LKSGT^550^ for *pbp*2x [71].

### Pneumococcal genotypic resistance

Pneumococcal β-lactam minimum inhibitory concentrations (MIC) were genotypically predicted using an analysis pipeline developed by the CDC [72, 73], and the results are reported in File S1. The MICs were interpreted using the Clinical and Laboratory Standards Institute (CLSI) guidelines and clinical breakpoints (https://clsi.org/). Genotypically predicted β-lactam resistance profiles and MICs using the pipeline are highly accurate and have been previously validated among some strains with available phenotypic data using CLSI breakpoints [72–75], and the pipeline has also been applied to pneumococcal isolates from the GPS Project [51, 52, 76].

### Horizontal genetic transfer analysis

The fastGEAR horizontal genetic transfer (HGT) software [44] was used to estimate and plot the amount of recent HGT in the *pbp* genes of *S. pneumoniae *, *S. mitis* and *S. oralis *. The sequence clusters of the *pbp*1a, *pbp*2b and *pbp*2x gene sequences were inferred using BAPS [66] within fastGEAR. The *pbp* sequence clusters inferred by BAPS that shared a common ancestry in at least 50 % of the sites were collapsed into recombination lineages using a hidden Markov model (HMM) [44]. The fastGEAR tool then detected recent recombination events between *pbp* recombination lineages using an HMM, and the origin of the recombinant sequence was then assigned to the *pbp* recombination lineage with the highest probability at that position. The statistical significance of recombination predictions was tested using a Bayes factor (BF) >1 for recent recombination events. The HGT analyses of *pbp1a, pbp2b and pbp2x* gene alignments were conducted individually using the default settings in fastGEAR. The HGT patterns between *pbp* lineages were visualised using ggplot2 [69] in R [70] and Cystoscape v 3.8.0 [77]. As an internal check of the *pbp* HGT analysis, we also investigated the extent of genetic transfer occurring at other streptococcal genes as a comparison. We investigated HGT among seven pneumococcal multi-locus sequence typing genes [78, 79], namely; shikimate dehydrogenase (*aroE*), d-alanine–d-alanine ligase (*ddl*), glucose-6-phosphate dehydrogenase (*gdh*), glucose kinase (*gki*), transketolase (*recP*), signal peptidase I (*spi*) and xanthine phosphoribosyltransferase (*xpt*). We also compared HGT among six other antibiotic resistance related genes, namely; dihydrofolate reductase (*folA*), dihydropteroate synthase (*folP*), DNA gyrase subunit A (*gyrA*), DNA gyrase subunit B (*gyrB*), DNA topoisomerase 4 subunit A (*parC*) and DNA topoisomerase 4 subunit B (*parE*).

### Statistical analyses

Mosaicism among the *pbp* genes, β-lactam susceptibility and active binding site motif status was compared among the pneumococci using the two-tailed Fisher’s exact test in GraphPad Prism v8.0 (GraphPad Software). The distribution of acquired *pbp* fragment sizes and the β-lactam MICs for pneumococcal strains with and without *pbp* mosaicism were assessed using the Mann–Whitney test. A *P*-value of less than 0.05 was considered to be statistically significant.

## Results

### Genomic identification of Streptococci

Following initial assessment of the quality and species assignment using KRAKEN v1.0 [53], we confirmed that 81 % (141 out of 174), 93 % (125 out of 135) and 100 % (1000 out of 1000), of *S. mitis *, *S. oralis* and *S. pneumoniae* respectively had been correctly identified (File S1). Two percent (3 out of 135) of *S. oralis* were reassigned as *S. mitis *, and 6 % (11 out of 174) of *S. mitis* were reassigned as *S. oralis *. We excluded one *S *. *oralis* and four *S *. *mitis* genomes among the confirmed species due to their having partial *pbp* genes from poorly assembled gene regions. In total, we used 1000 *S *. *pneumoniae *, 140 *S *. *mitis* and 135 *S *. *oralis* genomes in the HGT analysis.

### Population structure of global pneumococcal isolates

**Fig. 1.**
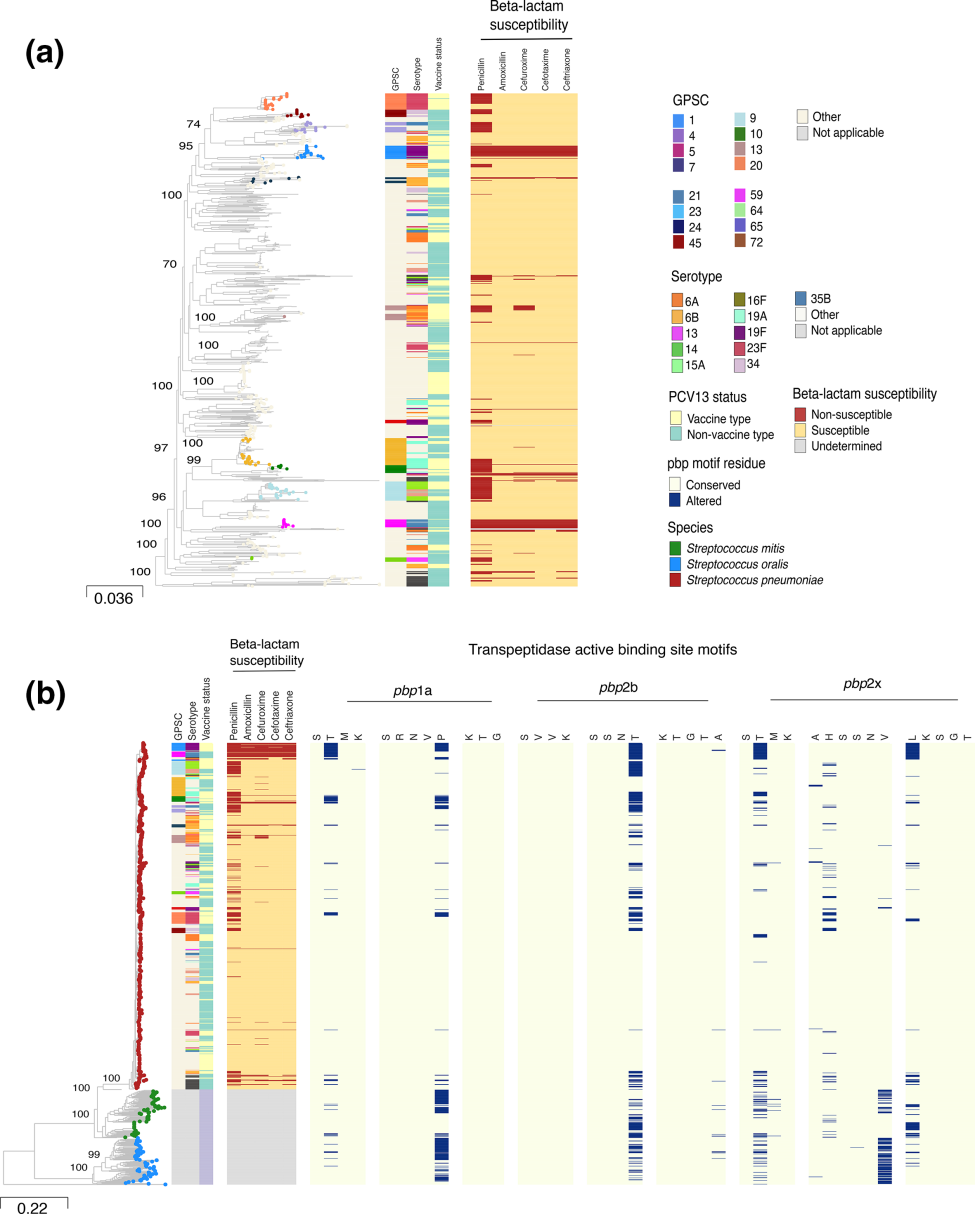
Core genome maximum likelihood phylogenies and transpeptidase active binding site analysis of *S. pneumoniae *, *S. mitis* and *S. oralis *. **a**) The maximum likelihood phylogeny of 1000 randomly selected carried pneumococcal strains from the Global Pneumococcal Sequencing Project datasets were constructed using core genome SNPs. The phylogeny demonstrates genetic similarity and diversity among the isolates. The strain metadata, namely GPSC, serotype, PCV13 status and β-lactam antibiotic susceptibility are shown. Support at the branches is indicated by the bootstrap values, and the tree was rooted at the mid-point. **b**) Core genome maximum likelihood phylogeny and active binding domain analysis of *S. pneumoniae, S. mitis* and *S. oralis* penicillin-binding proteins. On the left is a maximum likelihood tree based on core genome SNPs of *S. pneumoniae* (red), *S. mitis* (green) and *S. oralis* (blue). Pneumococcal strain metadata, namely GPSC, serotype, PCV13 status and β-lactam antibiotic susceptibility are shown. The larger panel on the right corresponds to the strains in the core genome phylogeny and shows the changes in *pbp*1a, *pbp*2b and *pbp*2x amino acid residues compared with the conserved *pbp* binding motifs of the β-lactam-susceptible *S. pneumoniae* R6 reference.

To establish the genetic diversity of the randomly sampled pneumococcal genomes, we characterised the strains through *in-silico* serotyping, sequence typing, lineage inference based on the GPSC nomenclature and prediction of β-lactam susceptibility. The core genome maximum likelihood phylogenetic tree of the pneumococcal isolates indicated that the pneumococcal isolates clustered in 176 GPSCs Fig. 1a. The random selection of 1000 pneumococcal genomes covered dominant, intermediate and rare GPSCs. Greater genomic diversity was observed among the 140 publicly-available *S. mitis* genomes (Fig. 1b), as previously described for a more limited dataset [80, 81].

We identified a total of 61 serotypes and 391 sequence types (STs) among the 1000 pneumococcal isolates (File S1). Pneumococcal conjugate vaccine 13 (PCV13) vaccine types (VTs) accounted for 43.9 % (439 out of 1000) of the isolates, which were spread across 81 GPSCs. A total of 36.7 % (161 out of 439) penicillin non-susceptible VT isolates with a penicillin MIC of greater than 0.06 µg ml^−1^ were identified (Table S1). We observed a high frequency of penicillin nonsusceptibility for serotypes 6A (19 out of 74), 6B (16 out of 56), 14 (27 out of 34), 19A (28 out of 52), 19F (32 out of 52) and 23F (31 out of 64) compared with other VTs (Table S2). PCV13 non-vaccine serotypes (NVTs) accounted for 51.0 % (510 out of 1000) of the isolates and were spread across 104 GPSCs, and 14.3 % (73 out of 510) were penicillin non-susceptible. Compared with other NVTs, we observed a high frequency of penicillin non-susceptibility among serotypes 13 (9 out of 15), 15A (13 out of 34), 34 (9 out of 27),and 35B (22 out of 36). Non-typeable pneumococci accounted for 5.1 % (51 out of 1000) of the isolates analysed and were spread across 17 GPSCs.

### *S. mitis* and *S. oralis* are reservoirs of altered *pbp* active binding site motifs and *pbp* genetic diversity

We investigated the *pbp* active binding site motifs in the TPD regions to identify species that are potential reservoirs of binding motif residues associated with pneumococcal β-lactam non-susceptibility. While the *pbp* binding site motif variants identified in *S. pneumoniae* confer reduced pneumococcal β-lactam susceptibility [28, 82–84], it has not been determined whether they confer reduced β-lactam susceptibility in *S. oralis* and *S. mitis *. Nonetheless, by comparing the active binding site motifs of the streptococcal genomes to motifs of the β-lactam-susceptible *S. pneumoniae* R6 genome (GenBank Accession GCA000007045.1), we identified amino acid changes in seven out of nine binding motifs among the streptococci (Fig. 1b). The domain regions used for the analysis were ^370^STMK^373^, ^428^SRNVP^432^, ^574^KTG^576^ for *pbp*1a, ^391^SVVK^394^, ^448^SSNT^451^ and ^620^KTGTA^624^ for *pbp*2b and ^337^STMK^340^, ^393^AHSSNV^398^ and ^546^LKSGT^550^ for *pbp*2x [85]. Overall, the majority of *S. pneumoniae* (699 out of 1000) had conserved *pbp* active binding motifs, whilst the vast majority of *S. mitis* (120 out of 140) and *S. oralis* (132 out of 135) had active binding motifs that are altered in the pneumococcus (Tables S3 and S4).

To demonstrate the relationship between *pbp* active binding motif status and β-lactam susceptibility in our global pneumococcal dataset [28, 82–84], we investigated the prevalence of altered *pbp* TPD residues among β-lactam susceptible and nonsusceptible pneumococci (Table S5). Among the 732 penicillin susceptible streptococcus pneumococci (PSSP), 91.1 % (667 out of 732) had conserved motifs and 8.9 % (65 out of 732) had altered motifs. While among the penicillin non-susceptible streptococcus pneumococci (PNSP), 11.4 % (30 out of 264) had conserved motifs and 88.6 % (234 out of 264) had altered motifs. There was a strong association between *pbp* motif status and penicillin susceptibility among the pneumococcal isolates (Fisher’s exact test *P*<0.0001).

**Fig. 2.**
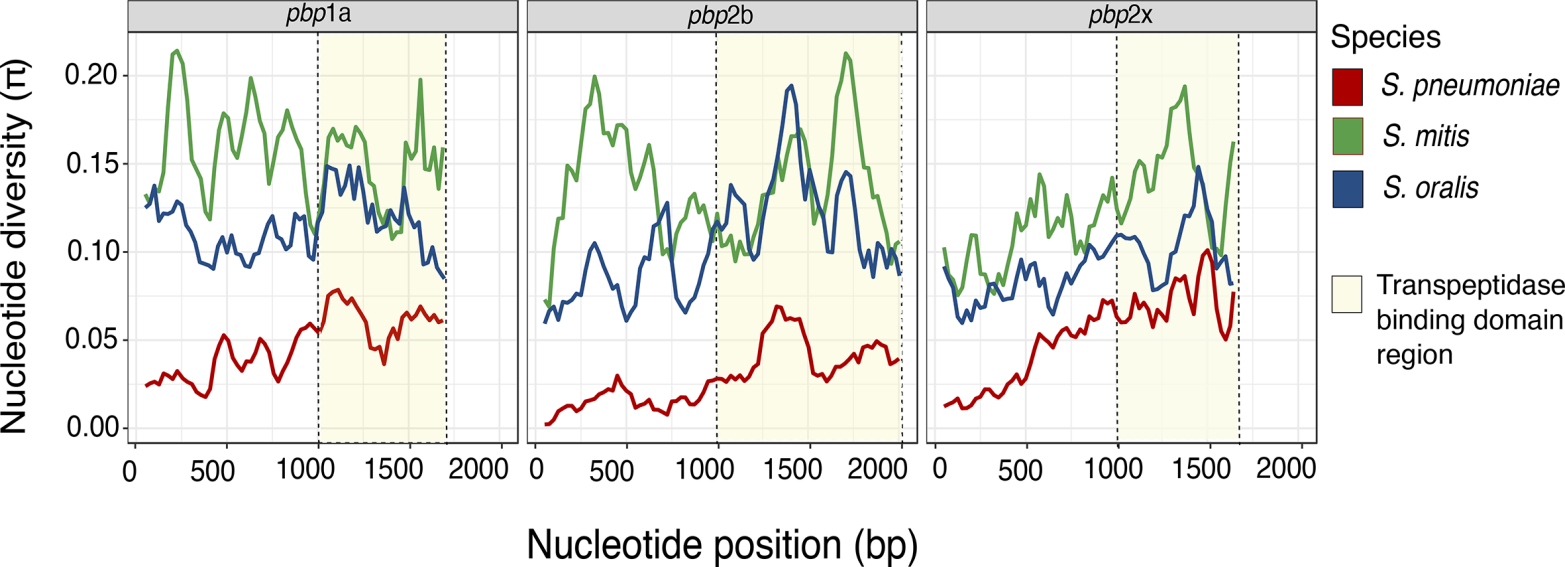
Nucleotide diversity sliding window analysis of *S. pneumoniae *, *S. mitis* and *S. oralis* penicillin-binding protein genes. The nucleotide analysis was applied to *S. pneumoniae *, *S. mitis* and *S. oralis pbp*1a (1737 bp), *pbp*2b (2049 bp) and *pbp*2x (1686 bp) alignments. Sliding window plot analysis showing nucleotide diversity (π) values across *pbp* sequences analysed for *S. pneumoniae *, *S. mitis* and *S. oralis *. A window size of 100 bp and a step size of 25 bp was used. The transpeptidase binding domain regions that harbour the active binding site motifs are highlighted.

We then explored *pbp* nucleotide diversity to quantify the amount of *pbp* gene variation among the different streptococcal species (File S1). *S. mitis* had the highest number of average nucleotide differences (K) for *pbp*1a (266.438), *pbp*2b (272.779) and *pbp*2x (207.083). Whilst *S. pneumoniae* had the least number of nucleotide differences for *pbp*1a (81.369), *pbp*2b (57.773) and *pbp*2x (86.710). A sliding window plot of nucleotide diversity (π) revealed that the streptococcal species have different patterns of nucleotide diversity throughout their sequences, but confirmed that *S. mitis* has a greater nucleotide diversity compared with *S. pneumoniae* and *S. oralis* across all *pbp* genes (Fig. 2).

### Extensive mosaicism occurs among the streptococcal *pbp* genes

We then aimed to determine the role that *S. mitis* plays as a pneumococcal genetic exchange partner for the pneumococcus compared with *S. oralis* and other pneumococci. We therefore investigated intraspecies and interspecies HGT of *pbp* gene fragments among *S. pneumoniae *, *S. mitis* and *S. oralis* using fastGEAR. We identified mosaicism among 36 % (361 out of 1000) of *S *. *pneumoniae *, 94 % (131 out of 140) of *S *. *mitis* and 79 % (106 out of 135) of *S *. *oralis* (Table 1) using the extracted and aligned *pbp1a* (1737 bp), *pbp2b* (2049 bp) and *pbp2x* (1686 bp) sequence alignments. *S. mitis* and *S. oralis* had a higher proportion of strains with recombined *pbp*1a, *pbp*2b and *pbp*2x genes compared with the pneumococcus. However, the undersampled non-pneumococcal species datasets do not provide a robust statistical signal to find potential recent HGT events into *S. mitis* or *S. oralis *. In relation to the previously described transpeptidase binding motif analysis (Fig. 1b), among the pneumococci with acquired *pbp* fragments 79.8 % (288 out of 361) strains had altered motifs and 20.2 % (73 out of 361) strains had conserved motifs. Whilst among the pneumococci that did not acquire *pbp* fragments, 2.0 % (13 out of 639) strains had altered motifs and 98.0 % (626 out of 639) of strains had conserved motifs. There was a strong association between *pbp* recombination and binding motif status among the pneumococci (Fisher’s exact test *P*<0.0001).

**Table 1.** Number and proportion of *S. pneumoniae *, *S. mitis* and *S. oralis* isolates with horizontally acquired *pbp* gene fragments identified using fastGEAR

Species	Number of strains	Number and proportion (%) of strains with recombined *pbp* genes			
		***pbp1a***	***pbp2b***	***pbp2x***	**Any *pbp* gene**
* S. pneumoniae *	1000	123 (12)	196 (20)	346 (35)	361 (36)
* S. mitis *	140	42 (30)	99 (71)	72 (51)	131 (94)
* S. oralis *	135	60 (44)	65 (48)	52 (39)	106 (79)

**Fig. 3.**
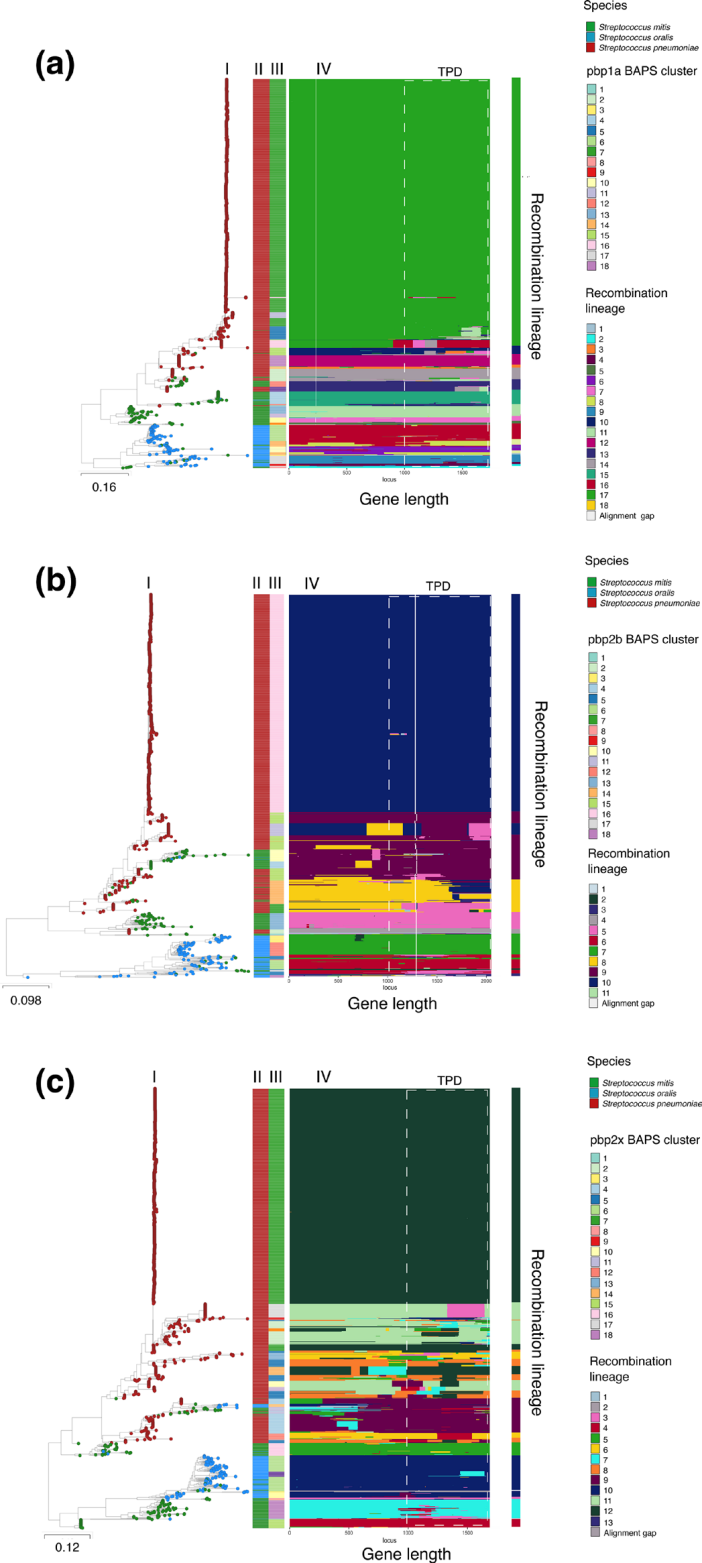
Horizontal genetic transfer analysis of *pbp1a*, *pbp2b* and *pbp*2x gene fragments among *S. pneumoniae *, *S. mitis* and *S. oralis* using the fastGEAR tool. On the left are **I**) maximum likelihood phylogenies based on **a**) *pbp*1a, **b**) *pbp*2b **c**) *pbp*2x gene alignments of *S. pneumoniae *, *S. mitis* and *S. oralis *.** II**) Species **III**) Bayesian analysis of population structure (BAPs) clusters of *pbp*1a, *pbp*2b and *pbp*2x determined using fastGEAR. **IV**) Recombination (lineage) block panel with HGT fragments identified by FastGEAR over the length of the *pbp* genes. Blocks of the same colour are proposed to be of the same origin (based on recombination lineages detected by fastGEAR). The transpeptidase binding domain regions that harbour the active binding site motifs are shown. The recombination lineages for the streptococcal *pbp* genes are shown next to the recombination lineage block panel.

To better understand streptococcal HGT at the level of *pbp* gene clusters, we investigated HGT among the *pbp* genes using maximum-likelihood phylogenies [48]. Phylogenetic analysis of the *pbp* genes revealed the sequences largely clustered by species (Fig. 3a–c I). *S. mitis* and *S. oralis pbp* sequences formed clusters with multiple deep branching lineages, whilst the majority of *S. pneumoniae* sequences were highly similar, less diverse and clustered together. The *pbp* genetic clusters identified using BAPS analysis supported the genetic clusters shown among the *pbp* phylogenies (Fig. 3a–c III). *S. pneumoniae *, *S. oralis* and *S. mitis pbp* sequences generally grouped together in several species-distinct *pbp* BAPS clusters. However, mixed species *pbp* sequences that clustered together by phylogeny were mostly observed between *S. mitis* and *S. pneumoniae,* indicating HGT. To infer recent HGT, *pbp* BAPS clusters that shared a common ancestry in at least 50 % of the sites were collapsed into *pbp* recombination lineages [44]. *S. pneumoniae pbp1a* sequences largely clustered together in a highly conserved recombination lineage 17 (Fig. 3a IV), but we identified evidence of HGT of *pbp*1A fragments from *S. mitis* (recombination lineage 7 and 11 origin) (Fig. 3a IV) and *S. oralis* (recombination lineage 16 origin) among pneumococcal strains in lineage 17. There is clear evidence of HGT of *pbp* fragments from *S. mitis* to *S. pneumoniae* that is consistent with reults reported previously [48]. Similarly, we also observed *pbp* fragment acquisition among pneumococcal recombination lineages for *pbp*2b (recombination lineages 8 and 10) and *pbp*2x (recombination lineages 8, 11 and 12). Acquired fragments predominately originated from *S. mitis* and other strains of *S. pneumoniae* (Fig. 3b, c IV). However, the undersampled non-pneumococcal genome datasets are a limitation and do not provide a robust statistical signal to accurately assess the extent and bi-directional HGT among the species.

**Fig. 4.**
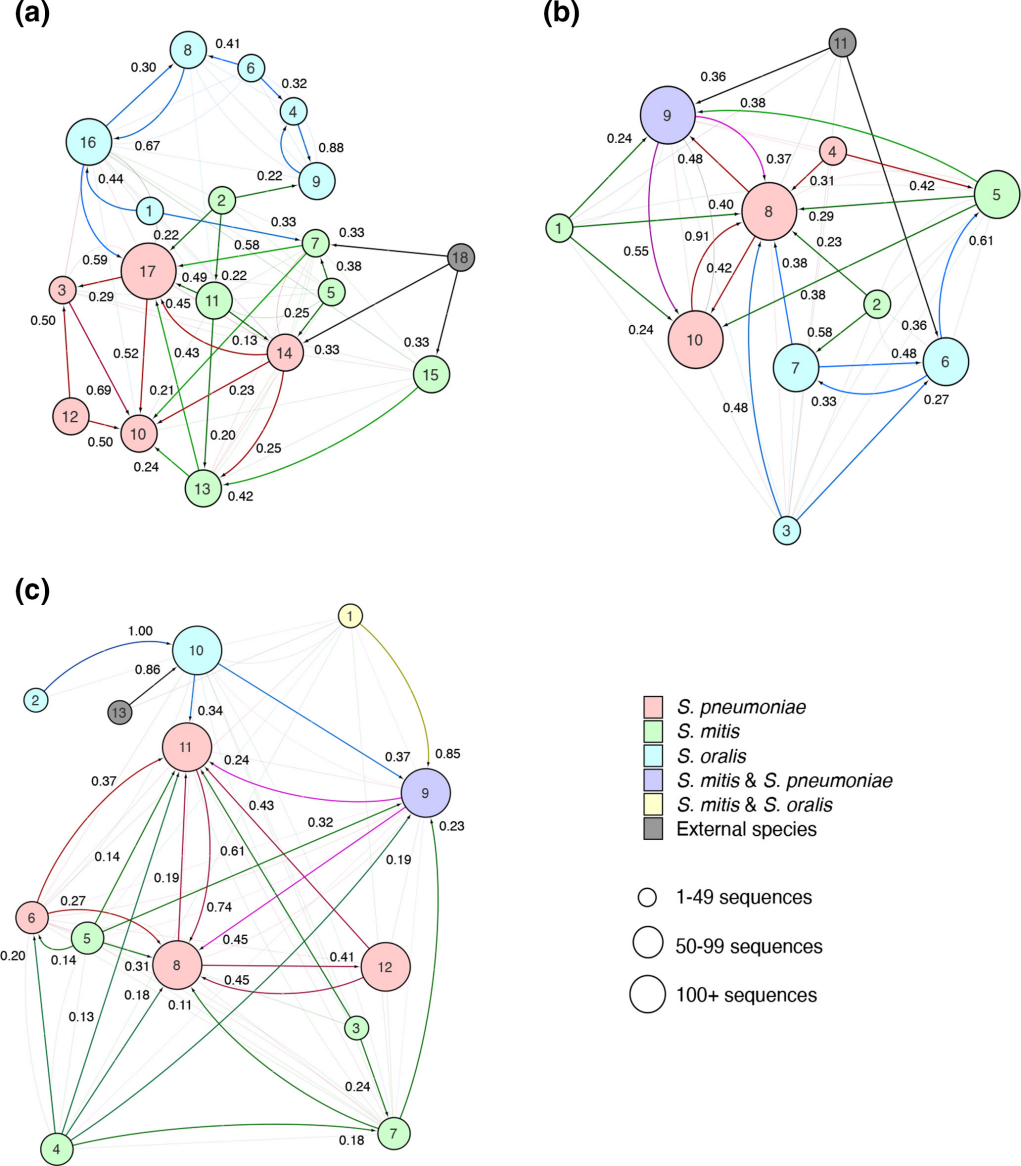
Admixture between streptococcal *pbp*1a, *pbp*2b and *pbp*2x fastGEAR lineages based on recent recombinations. Genetic interactions are shown between lineages identified by fastGEAR for a) *pbp*1a, b) *pbp*2b and c) *pbp*2x based on recent recombinations. Recombination lineages are coloured according to the dominant species within that *pbp* lineage, and the size is proportional to the number of *pbp* sequences within the lineage. Arrows indicate the proportion of sequence variation originating from the source lineage to *pbp* sequences of strains assigned to the target lineage. The highest proportions of variation originating from source lineages are indicated by bold arrows.

We then quantified the amount of HGT between the *pbp* recombination lineages to determine the extent to which each streptococcal species acted as a recombination donor (Fig. 4a–c). While the undersampled non-pneumococcal datasets remain a main limitation in assessing interspecies HGT, the results of this analysis indicate the proportion of *pbp* sequence variation originating from the source to strains assigned to the target lineage using a comprehensive dataset of publicly available *S. mitis* and *S. oralis* genomes. Similar to Fig. 3, pneumococcal *pbp* sequences mostly occupied *pbp*1a lineage 17 (Fig. 4a), *pbp*2b lineage 8 and 10 (Fig. 4b) and *pbp*2x gene lineage 8, 11 and 12 (Fig. 4c) in the admixture analysis of recent HGT. *S. mitis* was a greater source of *pbp* variation for the dominant pneumococcal *pbp* recombination lineages compared with *S. oralis *, however, we also observed intraspecies HGT among pneumococcal *pbp* lineages.

### Pneumococcal serotypes and genotypes with a high proportion of acquired *pbp* fragments

**Fig. 5.**
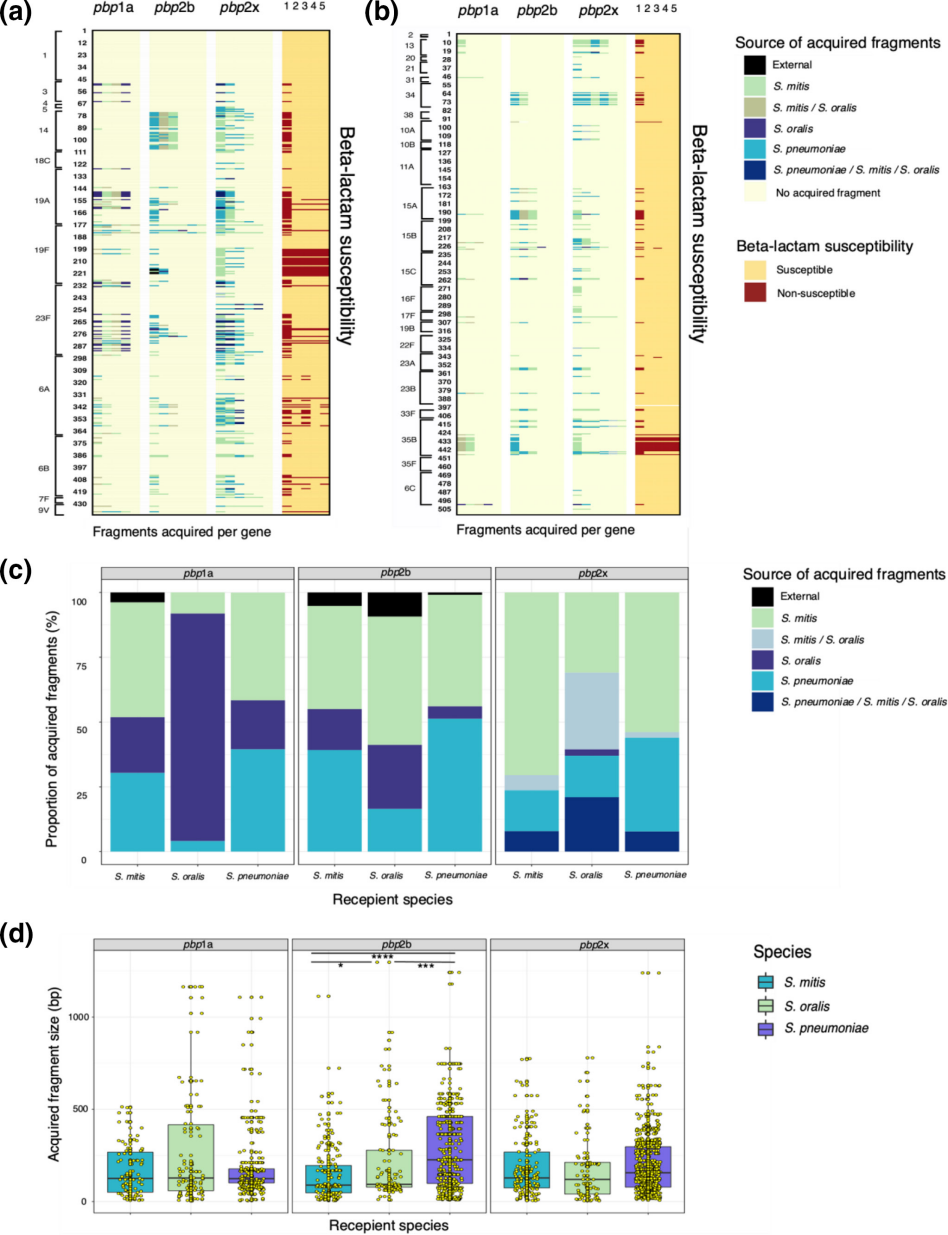
Analysis of *pbp* fragments acquired by pneumococcal serotypes, *S. mitis* and *S. oralis* strains. Distribution and size of acquired *pbp* fragments, *pbp* donor sources and β-lactam susceptibility among pneumococcal **a**) vaccine serotypes **b**) non-vaccine serotypes. Predicted pneumococcal β-lactam resistance is shown in the last columns where the antibiotics are; 1. Penicillin, 2. Amoxicillin, 3. Cefuroxime, 4. Cefotaxime, 5. Ceftriaxone. **c**) Donor source proportions of horizontally acquired *pbp* fragments among *S. pneumoniae *, *S. mitis* and *S. oralis* using recombination lineages determined by fastGEAR. **d**) Size distribution of acquired fragments in the *pbp* genes of *S. pneumoniae *, *S. mitis* and *S. oralis *.

We investigated the acquisition of *pbp* fragments in the context of pneumococcal VTs, NVTs, GPSCs and STs to identify pneumococci with greater interspecies acquisition of HGT fragments. Fig. 5a, b summarise the number and donor origins of fragments acquired by VTs and NVTs respectively, where 46.9 % (206 out of 439) of VTs and 22.4 % (114 out of 510) of NVTs acquired *pbp* fragments in at least one *pbp* gene. Among the non-typable pneumococci, 80.4 % (41 out of 51) had acquired fragments in at least one *pbp* gene. A high proportion of VTs with acquired *pbp* fragments included serotypes 6A (38 out of 74), 14 (30 out of 34), 19A (30 out of 52), 19F (35 out of 52) and 23F (39 out of 64) (Table S6), which were spread across various GPSCs (Table S7). NVTs with a high proportion of strains with acquired *pbp* fragments included serotypes 13 (9 out of 15), 16F (14 out of 29) and 35B (23 out of 36). Whilst non-typeable strains (41 out of 51) that received *pbp* fragments were from multiple GPSCs. Among the multiple STs, all 30 penicillin non-susceptible ST63 strains (Serotype 14) had acquired *pbp* fragments mostly from *S. mitis* and *S. pneumoniae* (Table S8). Serotype 35B ST558 strains (12 out of 13) were also among the top ranking STs with acquired *pbp* fragments from predominantly *S. mitis* and other *S. pneumoniae* strains (Table S8).

We established the donor sources of all acquired *pbp* fragments among each species, and the predominant donors for *S. pneumoniae* were *S. mitis* and other *S. pneumoniae* (Fig. 5c). Among the acquired *pbp* fragments we identified among the pneumococci, 125 (41.5 %), 187 (43.0 %) and 412 (53.9 %) of the respective *pbp1a*, *pbp2b* and *pbp2x* fragments were of *S. mitis* origin. Whilst 119 (39.5 %), 223 (51.3 %) and 277 (36.2 %) of the respective *pbp1a*, *pbp2b* and *pbp2x* fragments were from other strains of *S. pneumoniae *. *S. oralis* contributed to a much less extent as a donor for *S. pneumoniae pbp*1a (18.9%), *pbp*2b (4.8%) and *pbp*2x (0.0%) fragments. Mixed species with recombined *pbp* genes were also donors of *pbp* fragments, therefore for multiple acquired fragments the donor species may have been *S. mitis *, *S. oralis* or *S. pneumoniae *. We observed a greater degree of *pbp* fragment acquisition in the TPD region of pneumococcal *pbp* genes (83.4–94.4 %), which harbour the active binding motifs, compared with regions outside the TPD (Fig. S1). These results are consistent with those from previous studies [31–43]. We also analysed the sizes of the acquired *pbp* fragments (Fig. 5d), and the median sizes of the acquired fragments for the three species ranged from 105 to 133 bp for *pbp1a*, from 206 to 229 bp for *pbp2b* and from 147 to 182 bp for *pbp2x*. There was no significant difference in the size distribution of *pbp* fragments acquired across *pbp*1a and *pbp*2x genes among the species. However, we observed a significant difference in the size distribution between all species for fragments acquired in the *pbp*2b gene (*P*<0.0455), with *S. pneumoniae* having acquired larger *pbp* fragments compared with *S. mitis* and *S. oralis *.

**Fig. 6.**
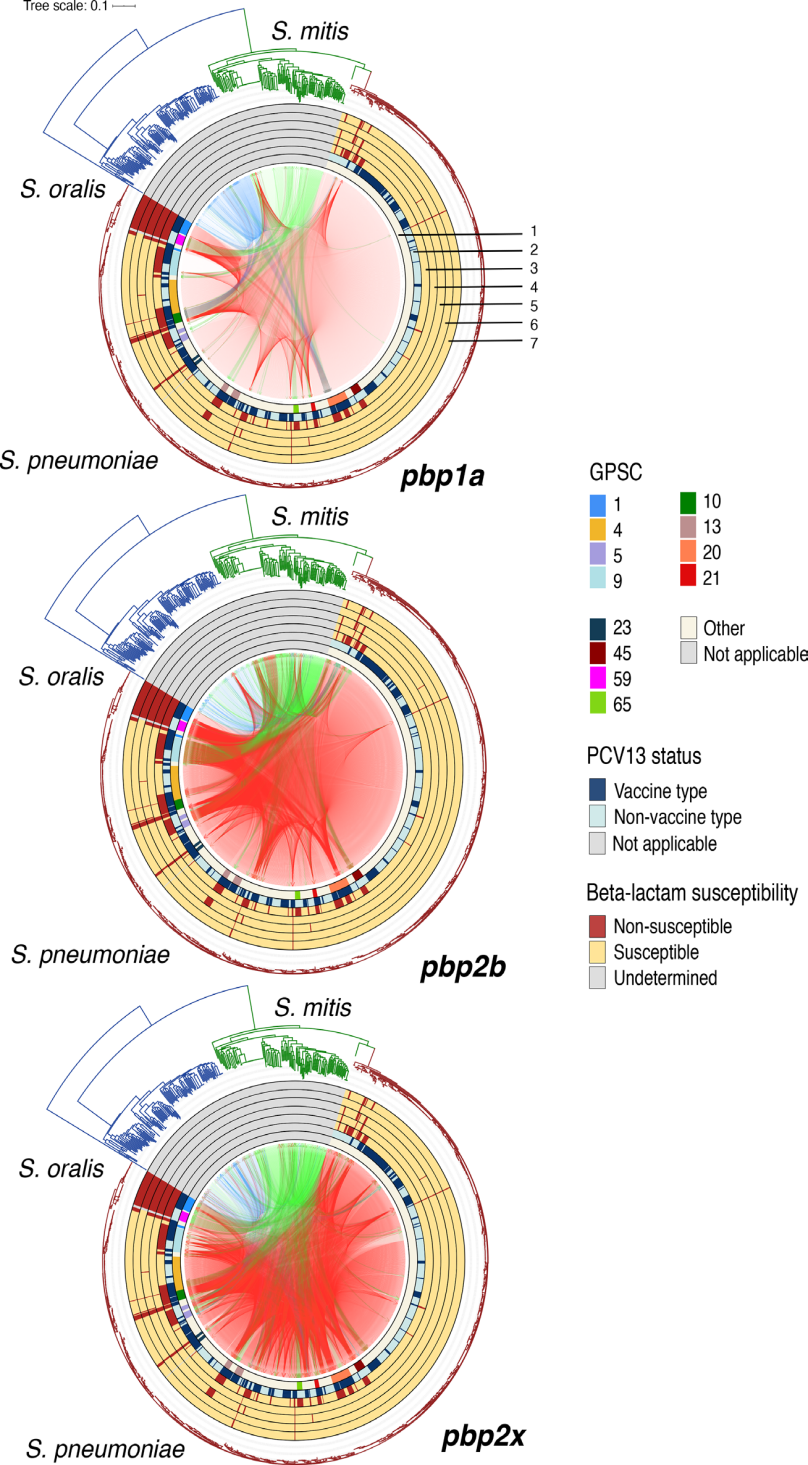
Genetic interactions among *S. pneumoniae *, *S. mitis* and *S. oralis pbp* genes. Maximum-likelihood trees based on SNPs from the core genome alignment of *S. pneumoniae, S. mitis* and *S. oralis* are shown in the outermost ring. Connections between donors and recipients of *pbp* fragments based on fastGEAR recombination lineages are shown in the centre for **a**) *pbp*1a, **b**) *pbp*2b and **c**) *pbp*2x, where arrows depict recipients of *pbp* fragments. From the inner ring: Global Pneumococcal Sequence clusters. Next ring: PCV13 status. Next five rings: Penicillin, amoxicillin, cefuroxime, cefotaxime and ceftriaxone susceptibility. *S. mitis* and *S. oralis* are coloured grey for features that included GPSC, PCV13 status and β-lactam susceptibility.

We then investigated the acquisition of *pbp* fragments in context of GPSCs, to determine if particular genotypes had a greater interspecies acquisition of HGT fragments. Overall, the top ranking GPSCs with acquired *pbp* fragments by proportion of strains were GPSC10 (16 out of 16), GPSC59 (16 out of 16), GPSC9 (37 out of 39), GPSC5 (16 out of 17), GPSC1 (23 out of 27), GPSC20 (22 out of 23) and GPSC45 (9 out of 15) (Fig. S2). Table S7 shows the associated serotypes of the top ranking GPSCs with acquired *pbp* fragments by proportion of strains. A high-resolution view of intraspecies and interspecies HGT of *pbp* fragments is summarised in Fig. 6, where evidence of *S. mitis* to *S. pneumoniae* HGT is illustrated for the top-ranking *S. pneumoniae* GPSCs.

### Interspecies *pbp* HGT occurs among intermediate and rare pneumococcal GPSCs

Our original analytical approach was to assess the epidemiological importance of *pbp* HGT between *S. mitis *, *S. oralis* and *S. pneumoniae* genotypes. However, the epidemiological sampling approach may have underestimated the potential for rarer pneumococcal genotypes to acquire *pbp* gene fragments. We have therefore used a phylogenetically informed approach to obtain previously undersampled intermediate and rare sequence clusters (Table S9), maximising genetic diversity in the HGT analysis. Using the GPS dataset of 13 454 strains [86], we randomly selected 1–2 genomes per intermediate and rare GPSC, as a majority of rare GPSCs (197 out of 371) are represented by one strain each [86]. We obtained 809 pneumococcal genomes which represented all 132 intermediate and 371 rare GPSCs in total (Table S9) and these genomes are listed in File S1. The results of this additional analysis indicate that HGT occurs among the *pbp* genes of 82 out of 264 (31 %) intermediate and 134 out of 545 (25 %) rare GPSC strains (Figs S3–S5) and that there is evidence of *S. mitis* to *S. pneumoniae* HGT for more than 50 % of acquired fragments across each *pbp* gene (Fig. S6). The results obtained in this additional analysis are therefore similar to those from the epidemiologically sampled dataset, specifically that a high proportion serotype 6A, 6B, 19A, 19F, 23F and non-typeable strains have acquired *pbp* fragments (Fig. S7). However, the phylogenetically informed approach highlights that HGT occurs even among intermediate and rare genotypes.

### Decreased β-lactam susceptibility among pneumococci with acquired *pbp* fragments

**Fig. 7.**
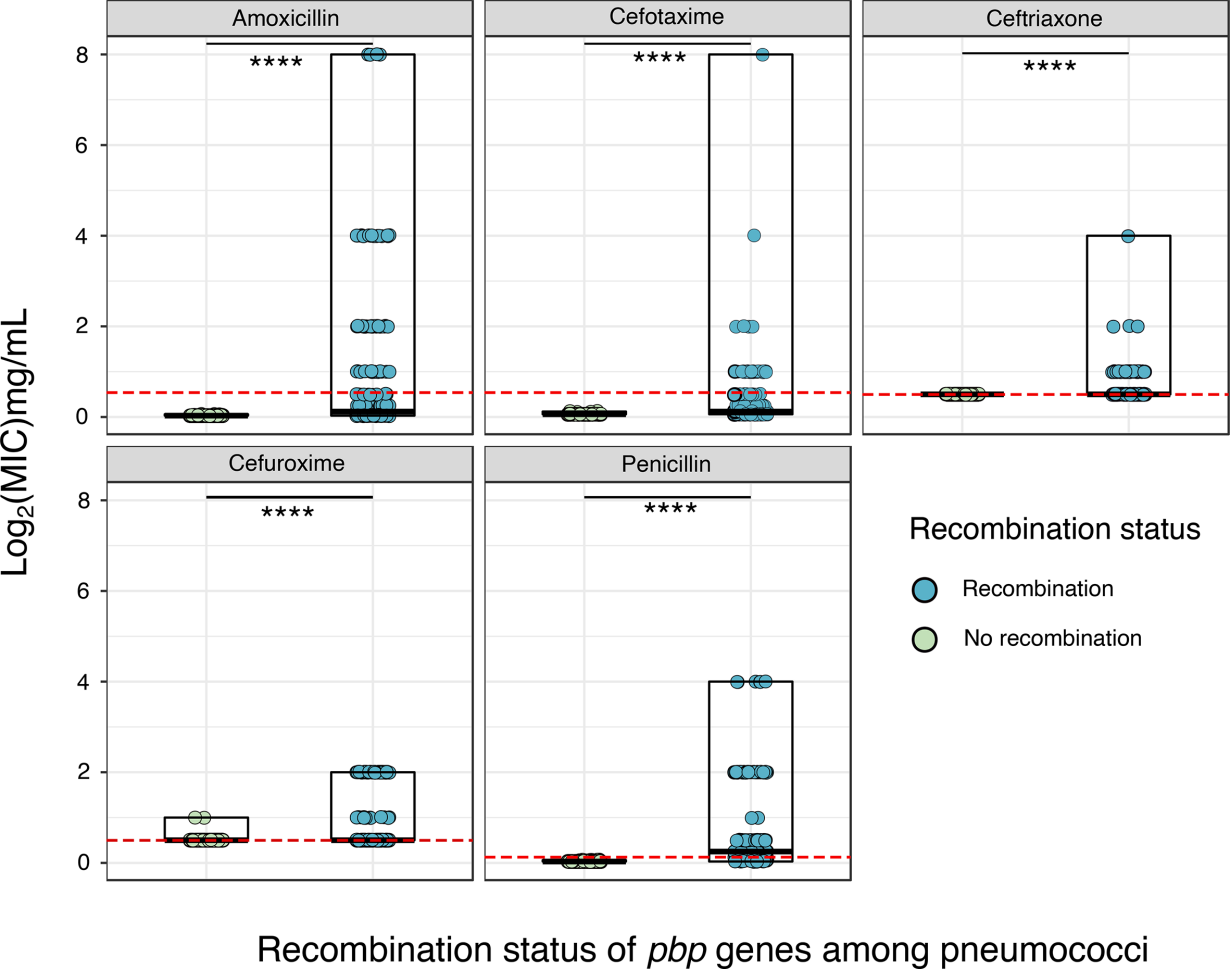
Distribution of β-lactam MIC values for pneumococcal strains with and without acquired *pbp* fragments. Distribution of penicillin, amoxicillin, cefuroxime, cefotaxime and ceftriaxone MICs values for pneumococci with and without acquired *pbp* fragments. The red horizontal dotted lines mark CLSI clinical breakpoints for non-meningitis disease, and all pneumococci with MIC values below the line are susceptible and pneumococci with MICs above the line are non-susceptible (intermediate or resistant). The thick black horizontal line marks the median MIC for each group, and the rectangle shows the range of MIC values.

We used the epidemiologically sampled pneumococcal dataset to determine if there is an association between *pbp* fragment acquisition and β-lactam susceptibility. Fig. 7 shows the distribution of β-lactam MIC among the pneumococci with and without acquired *pbp* fragments. Pneumococcal isolates which acquired *pbp* fragments (361 out of 1000) had a higher median MIC and wider MIC range for penicillin (0.25 µg ml^−1^, 0.03125–4 µg ml^−1^), compared with pneumococci without acquired fragments (0.03 µg ml^−1^, 0.03125–0.0625 µg ml^−1^). Similarly, higher median MIC values for amoxicillin (0.125 µg ml^−1^) and cefotaxime (0.125 µg ml^−1^) were observed for pneumococci which acquired *pbp* fragments, compared to isolates without acquired fragments (amoxicillin - 0.03 µg ml^−1^, cefotaxime - 0.0625 µg ml^−1^). Pneumococci which acquired *pbp* fragments also had wider MIC ranges for amoxicillin, cefuroxime, and ceftriaxone compared with pneumococci without acquired fragments.

The mean MICs for penicillin, amoxicillin, cefuroxime, cefotaxime and ceftriaxone were significantly different (*P*<0.0001) between pneumococci with and without acquired *pbp* fragments. Pneumococcal isolates which did not acquire *pbp* fragments (639 out of 1000) were fully susceptible to penicillin (MIC <0.06 mg ml^−1^), amoxicillin (MIC <0.5 mg ml^−1^), cefotaxime (MIC <0.5 mg ml^−1^) and ceftriaxone (MIC <0.5 mg ml^−1^). The results we obtained showing the relationship between *pbp* recombination and reduced penicillin susceptibility has previously been demonstrated using fastGEAR [44], and the relationship between mosaic *pbp* genes and reduced susceptibility in the pneumococcus is also supported by earlier work [31–43].

### HGT among MLST house-keeping and other antibiotic resistance genes

As an internal check of HGT, we also investigated HGT among seven housekeeping genes used for pneumococcal MLST (Fig. S8) and six other genes associated with AMR (Fig. S9). We applied the internal check on the epidemiologically sampled pneumococcal dataset. *S. pneumoniae* had the least proportion of strains with horizontally acquired fragments in the MLST genes (0–9 %) compared with *S. mitis* (2.9–20 %) and *S. oralis* (0–20.1 %) (Table S10). Unlike other MLST genes, the *ddl* gene had pneumococcal sequences scattered across the entire phylogeny (Fig. S8b), predominately among *S. mitis ddl* BAPS clusters, indicating HGT. Our observation is supported by earlier work that has described the *pbp2B–ddl* gene region as an observed recombination hotspot [40], presumably as a function of selectable *pbp2B* alleles. Among the AMR genes, we observed a high degree of mosaicism among *S. pneumoniae* for *folA* (19.8%) and *folP* (28.1%) (Table S10), which is consistent with previous results [30]. Similarly, we identified a high degree of HGT among the folate genes for *S. mitis* (*folA*, 16.4%; *folP*, 51.4%) and *S. oralis* (*folP*, 21.1%). There was minimal HGT across genes that confer resistance to fluoroquinolone antibiotics for the pneumococcus, with 0–1.9 % of pneumococcal isolates having acquired fragments in *gyrA*, *gyrB*, *parC* and *parE* genes. Earlier work similarly supports that interspecies recombination contributes minimally to fluoroquinolone resistance in the pneumococcus [87]. However, we observed a higher proportion of strains with acquired fragments among fluoroquinolone resistance genes for *S. mitis* (5.1–31.4 %) and *S. oralis* (8.3–46.6 %).

## Discussion

We demonstrate extensive HGT among the *pbp* genes of frequently carried pneumococcal serotypes and GPSCs associated with long carriage duration, high recombination rates and reduced β-lactam susceptibility. Pneumococcal serotypes typically carried for long durations in the human respiratory tract [88, 89] that include serotype 6A, 13, 14, 16F, 19A, 19F, 23F and 35B were the highest ranking serotypes with acquired *pbp* fragments in our analyses. *S. pneumoniae* isolates mostly associated with reduced β-lactam susceptibility acquired genetically diverse *pbp* gene fragments predominately from *S. mitis,* which builds upon earlier [31–43] and more recent studies on HGT involving *pbp* genes [48], that provide clear evidence of HGT from *S. mitis* to *S. pneumoniae *. Together these data indicate that the *S. mitis* are an important reservoir of *pbp* genetic diversity that may facilitate the acquisition of AMR, particularly amongst commonly carried pneumococcal VTs and NVTs associated with expanding and emerging pneumococcal lineages.

HGT between pneumococcal and *S. mitis* strains is more likely to introduce more variation in pneumococcal *pbp* genes that could alter β-lactam susceptibility compared with pneumococcal HGT with *S. oralis* or other strains of *S. pneumoniae *. Our results indicate that *S. mitis* is an important donor of genetically diverse *pbp* mosaic fragments to pneumococcal strains compared with *S. oralis *, which provides further evidence supporting the role of *S. mitis* as a major genetic exchange partner with the pneumococcus [31–43, 81, 90]. It has been established that *S. mitis* is genetically more diverse when compared with the pneumococcus [80, 81], therefore this may explain the greater sequence diversity of the *pbp,* MLST and other AMR genes observed among *S. mitis* in comparison to the pneumococcus. *S. oralis* was less of a pneumococcal HGT partner, which is supported by existing evidence that shows that *S. oralis* is more distantly related to the pneumococcus than *S. mitis* [80, 81, 90], and recombination events are known to decrease exponentially with genetic distance between donor and recipient strains [91]. Pneumococcal intraspecies HGT was also common in our analysis, and it has been suggested that recombination occurs more frequently within than between species [92, 93]. However, in our analysis *S. mitis* had the highest nucleotide diversity across all *pbp* genes compared with *S. pneumoniae* and *S. oralis *. At the amino acid level, *S. mitis* also had a majority of *pbp* motifs that are associated with reduced β-lactam susceptibility in the pneumococcus. In support of *S. mitis* as a source of genetic variation, the species has been shown to account for most of the *pbp*2x sequence diversity observed among *S. pneumoniae* and other viridans group streptococci [94]. The role of selection at the *pbp* genes that have among the highest rates of recombination in *S. pneumoniae* is a considerable challenge that was not addressed in our work. For example, alleles associated with resistance could arise by mutation or HGT. Alleles associated with resistance could also possibly arise from mutations acquired after recent HGT has occurred, and these alleles could also then be recently acquired by other pneumococci. Therefore, although selection may play a role in pneumococcal beta-lactam resistance, it may be difficult to untangle selection in the context of recombination. Indeed, it has been established that the *pbp* genes are recombination hotspots in the pneumococcus [13, 30], and that beta-lactam resistance has been driven by recombination at these loci [31–43, 46–48].

As one of the predominant streptococci in the oropharynx [49, 50], *S. mitis* has served as the most important HGT partner for commonly carried pneumococcal serotypes, which then appear to frequently serve as gene donors themselves of selectable non-pneumococcal *pbp* gene alleles. We found that pneumococci with a high proportion of horizontally acquired *pbp* fragments were among commonly carried serotypes including 6A, 13, 14, 16F, 19A, 19F, 23F and 35B [10, 11, 95, 96], which have been previously shown to be associated with higher rates of penicillin resistance compared with other pneumococcal serotypes [11, 97]. The serotypes and related GPSCs with a high proportion of acquired *pbp* fragments in our analyses are known to have higher proportions of observed recombinant sequences [51, 89], which has been linked to long duration of carriage [89]. Commonly carried serotypes with long carriage duration and a higher propensity for genetic exchange could be exposed more frequently to *S. mitis* in the naso-oropharynx, where 51–76 % *S *. *mitis* carriage prevalence among healthy individuals has been described [49, 50]. Longer and more frequent exposure between *S. mitis* and the pneumococcus may provide greater opportunity for HGT events between the species. We propose that in high burden regions where recombination between pneumococcal strains is likely to be high [98], co-colonising *S. mitis* may be an important donor of genetic material for the pneumococcus which may contribute to antibiotic resistance [12, 99].

HGT between *S. mitis* and *S. pneumoniae* may be particularly important in the context of pneumococcal vaccine escape, expansion of NVTs and the emergence of pneumococcal AMR. Serotype 35B ST558, an increasing cause of IPD in the post-PCV introduction era in the USA [17] was a common recipient of *pbp* fragments in our analysis that was resistant to penicillin, amoxicillin, cefuroxime, cefotaxime and ceftriaxone. Over the past 20 years in the USA the increase in IPD caused by penicillin non-susceptible serotype 35B was due to primarily the ST558 lineage, which was demonstrated to be a frequent recombination donor for other pneumococci [17]. This is an important finding in the context of expanding NVT lineages in the post-PCV era, as lineages such as ST558 may have a greater propensity for HGT with commensal species and other pneumococci. Thus, ST558 could potentially disseminate resistance determinants obtained from *S. mitis* to other pneumococcal lineages. Non-typeable pneumococci, strains that do not express the pneumococcal capsule, were also recipients of *pbp* fragments, and previously pneumococcal recombination studies have highlighted these pneumococci as dominant recombination donors and recipients [30]. Thus non-typeable pneumococci could also potentially disseminate *pbp* fragments obtained from *S. mitis* to other pneumococcal lineages [100]. We observed a high proportion of penicillin resistant ST63 (Serotype 14/15A) PMEN25 strains from multiple geographical locations that acquired *pbp* fragments from predominantly *S. mitis* through HGT. Genetic promiscuity is known to have contributed to other highly successful resistant clones, such as the PMEN1, which has evaded vaccine pressure multiple times and acquired drug resistance [12, 101, 102]. Previous comparative analyses of *pbp*2x genes have indicated that mosaic genes of PMEN1 have been observed among global viridans species [94]. Therefore, *S. mitis* may have played an important role in the evolution of MDR PMEN lineages, such as the PMEN25 clone, and *S. mitis* may continue to facilitate the emergence of β-lactam resistance among pneumococcal clones.

Our findings were limited by the number of publicly available *S. mitis* and *S. oralis* genomes used for the HGT analysis, which may have resulted in an underestimation of the extent of HGT with the pneumococcus. The under sampled non-pneumococcal datasets also do not provide a robust statistical signal to fully assess whether one species acts as more of a HGT partner compared with another and to assess the extent of recent HGT among *S. mitis* and *S. oralis *. The *S. mitis* and *S. oralis* genomes were also not all sampled from the same region. Although further sequencing of systematically collected commensal streptococci is warranted, our analysis offers a substantial insight into the genetic interactions between the streptococci with the publicly available genomic data. We did not include other distantly related species of the genus *Streptococcus *, such as *Streptococcus infantis *, in the analysis due to limited publicly available genomic data, but recent data has indicated that *S. infantis* shares similar capsular genes with the pneumococcus [103]. Therefore, it is possible that *S. infantis* may be an important pneumococcal HGT partner involving other genes.

Although the *in silico* HGT analysis could potentially introduce artefacts, we investigated HGT at the conserved MLST genes and we confirmed minimal exchange among the loci. We also investigated HGT among non-*pbp* AMR genes and similarly identified folate biosynthesis genes as recombination hotspots as described in earlier work [13, 30], thus providing further support for the extensive *pbp* HGT observations in our analysis. Although we established specific dominant GPSC genotypes with a greater degree of HGT than other GPSCs, we confirmed that HGT also occurs among intermediate and rare GPSC strains.

In conclusion, this large-scale genomic analysis demonstrates *S. mitis* to be an important donor and reservoir of *pbp* diversity that was associated with reduced β-lactam susceptibility in carried pneumococci. Expanding pneumococcal lineages with increased opportunities for recombination through prolonged carriage were among the recipients of *pbp* fragments from *S. mitis* with the potential to disseminate resistance determinants to the wider pneumococcal population. Investigating the mechanisms of genetic transfer through genomic surveillance of the pneumococcus and closely related commensal species is necessary in understanding the evolution of β-lactam resistance among pneumococcal lineages that escape clinical interventions. As pneumococcal vaccine programmes mature, probably shifting the pneumococcal population structure, it will be important to monitor the influence of HGT of antimicrobial resistance determinants from commensal streptococci such as *S. mitis *.

## Supplementary Data

Supplementary material 1

Supplementary material 2
