# Supplementary material for: Streptococcus pneumoniae serotypes that frequently colonise the human nasopharynx are common recipients of penicillin-binding protein gene fragments from Streptococcus mitis
Source: Microb Genom. 2021 Sep 22;7(9):000622. doi: 10.1099/mgen.0.000622 (PMC8715442; doi:10.1099/mgen.0.000622)
Supplement: Supplementary material 1 [file mgen-7-0622-s001.pdf]

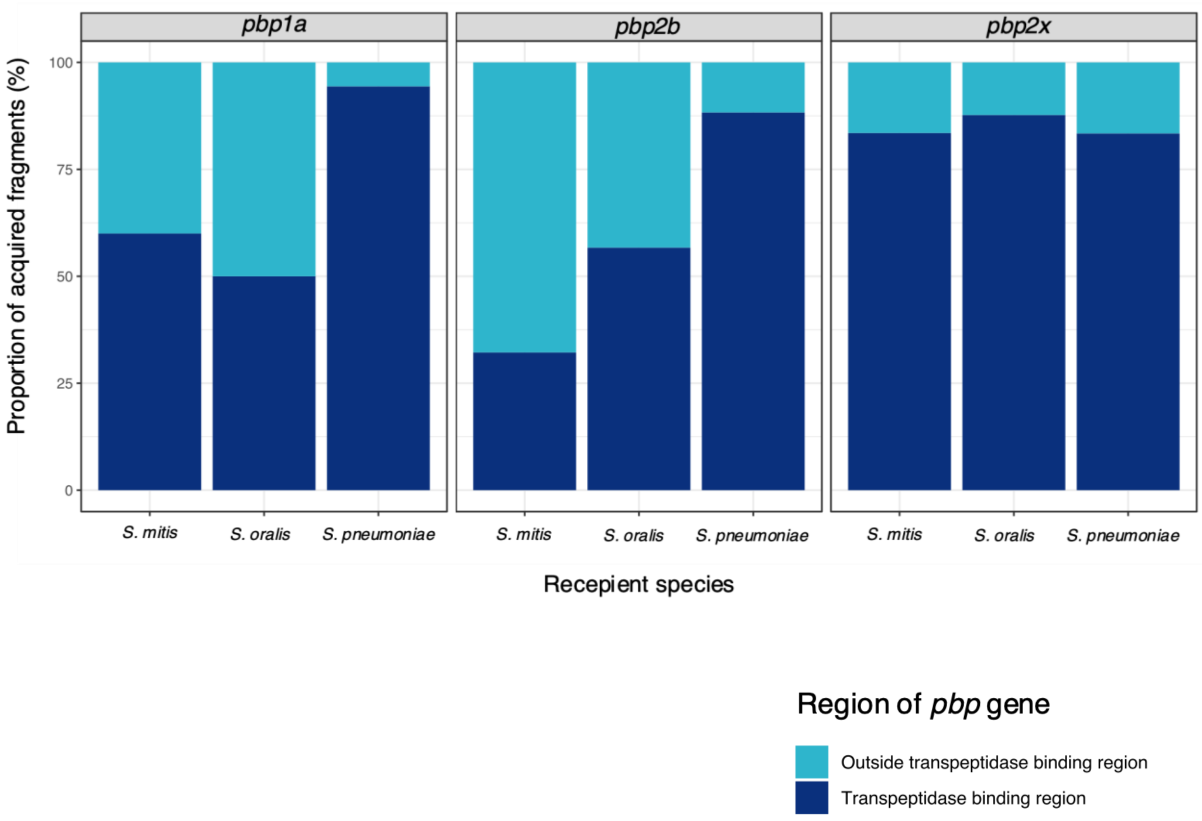

S1 Fig: **Proportion of *pbp* fragments acquired by *S. pneumoniae*, *S. mitis*, and *S. oralis* within and outside the transpeptidase binding domain region (TDR) of the *pbp* genes.**

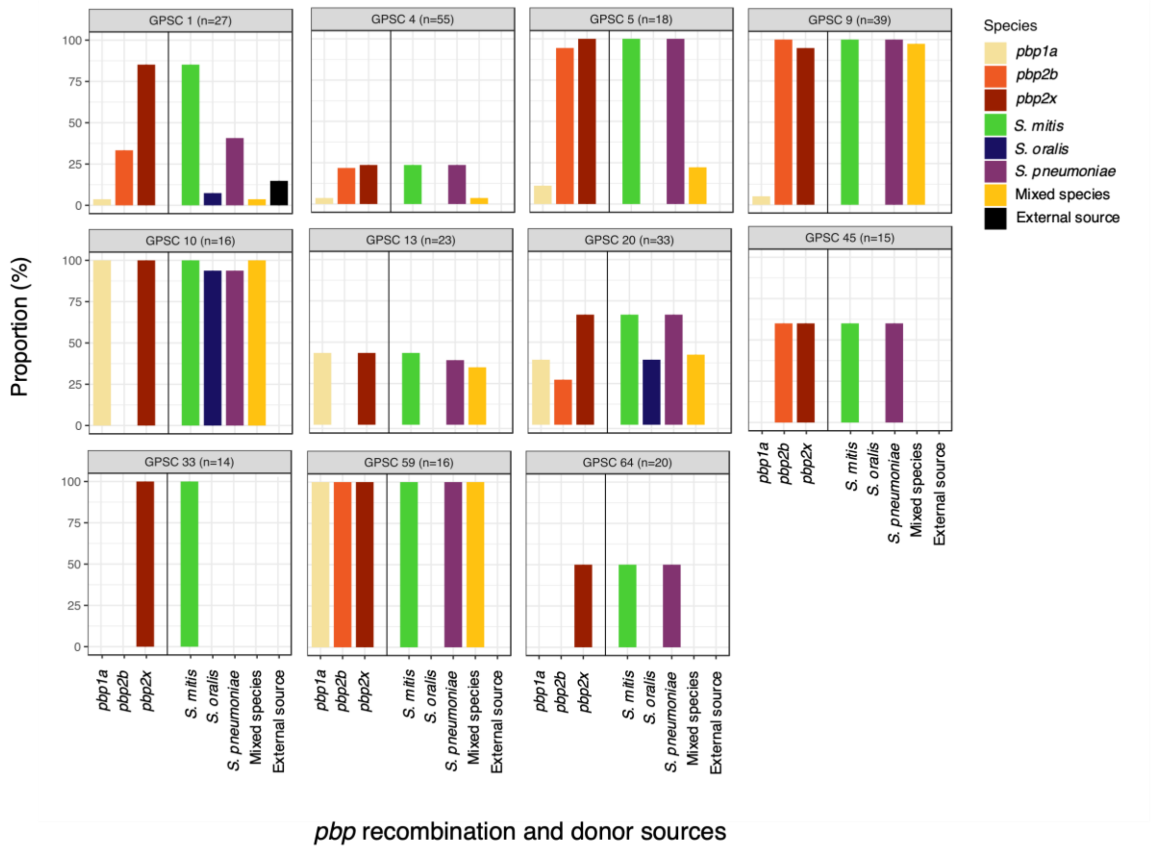

S2 Fig: Top ranking GPSCs with acquired *pbp* fragments by proportion of strains. The top ranked GPSCs with acquired *pbp* fragments are shown, and for each GPSC the panel on the left shows the proportion of strains with acquired fragments in *pbp*1a, *pbp*2b, and *pbp*2x. Whilst the right panel shows the proportion of strains with acquired *pbp* fragments from *S. mitis*, *S. oralis*, *S. pneumoniae*, mixed species, or external sources.

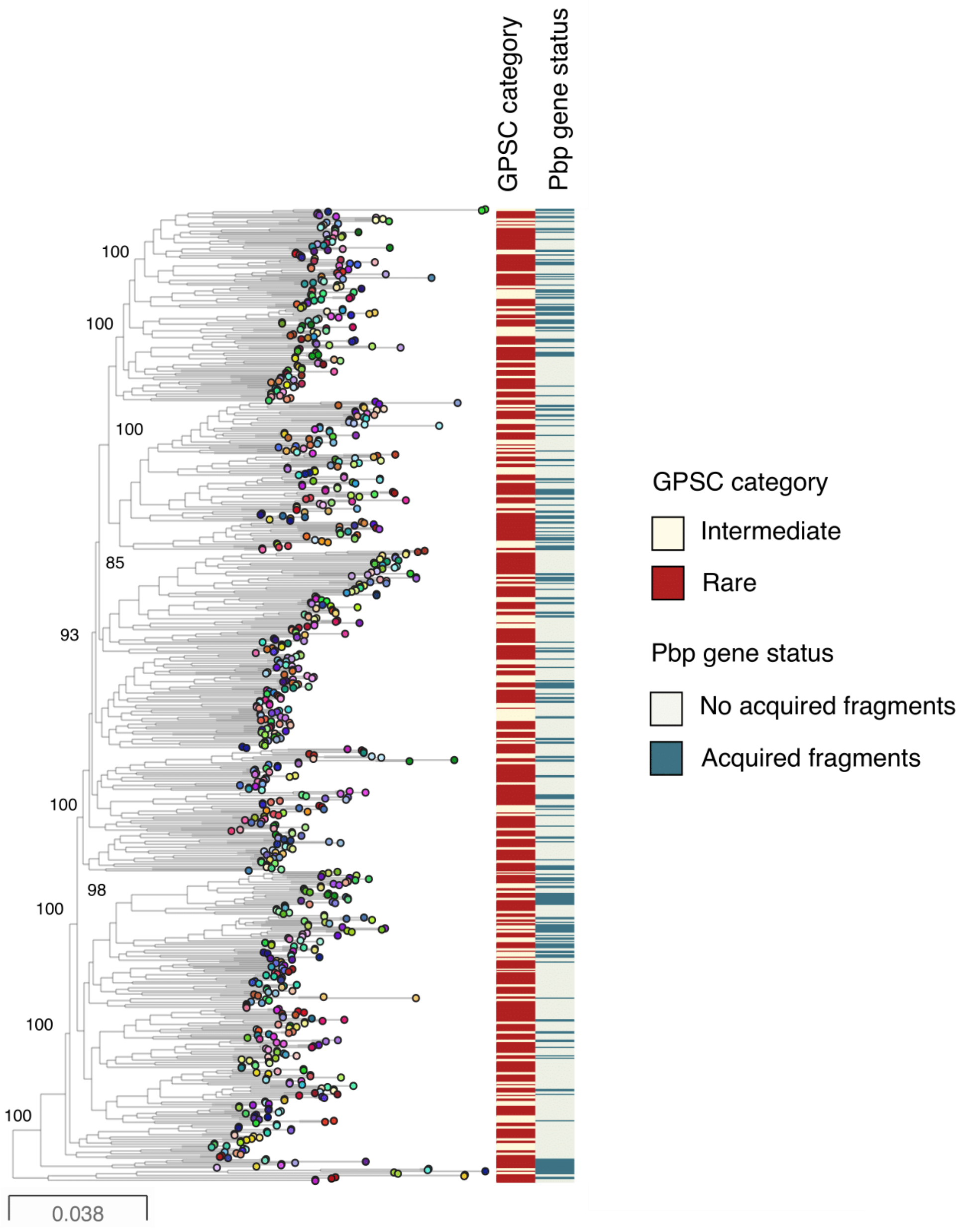

S3 Fig: Core genome maximum likelihood phylogeny of randomly sampled intermediate and rare GPSC strains. The maximum likelihood phylogeny of 809 randomly selected intermediate (264/809) and rare (545/809) pneumococcal GPSC strains from the Global Pneumococcal Sequencing Project datasets were constructed using core genome SNPs. The phylogeny demonstrates genetic similarity and diversity among the isolates. The strain metadata, namely GPSC category, evidence of acquired *pbp* fragments, and penicillin susceptibility are shown. Support at the branches is indicated by the boot strap values, and the tree was rooted at the mid-point.

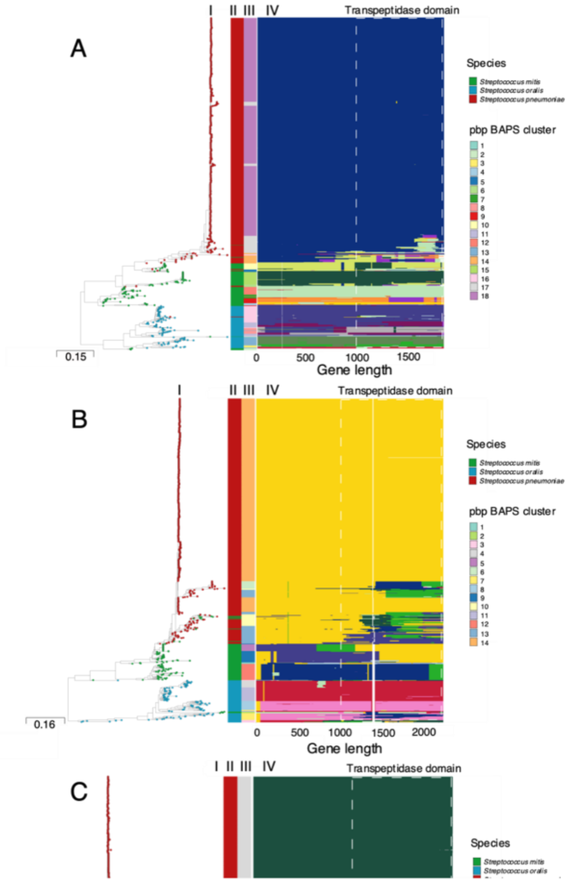

S4 Fig: **Horizontal genetic transfer analysis of *pbp1a*, *pbp2b*, and *pbp*2x gene fragments among intermediate and rare *S. pneumoniae* GPSC strains, *S. mitis*, and *S. oralis* using the fastGEAR tool**. **I**) On the left are maximum likelihood phylogenies based on **A**) *pbp*1a, **B**) *pbp*2b **C**) *pbp*2x gene alignments of *S. pneumoniae*, *S. mitis*, and *S. oralis.* **II**) Species **III**) Bayesian analysis of population structure (BAPs) clusters of *pbp*1a, *pbp*2b, and *pbp*2x determined using fastGEAR. **IV**) Recombination (lineage) block panel with HGT fragments identified by FastGEAR over the length of the *pbp* genes. Blocks of the same colour are hypothesised to be of the same origin (based on recombination lineages detected by fastGEAR). The transpeptidase binding domain regions that harbour the active binding site motifs are shown.

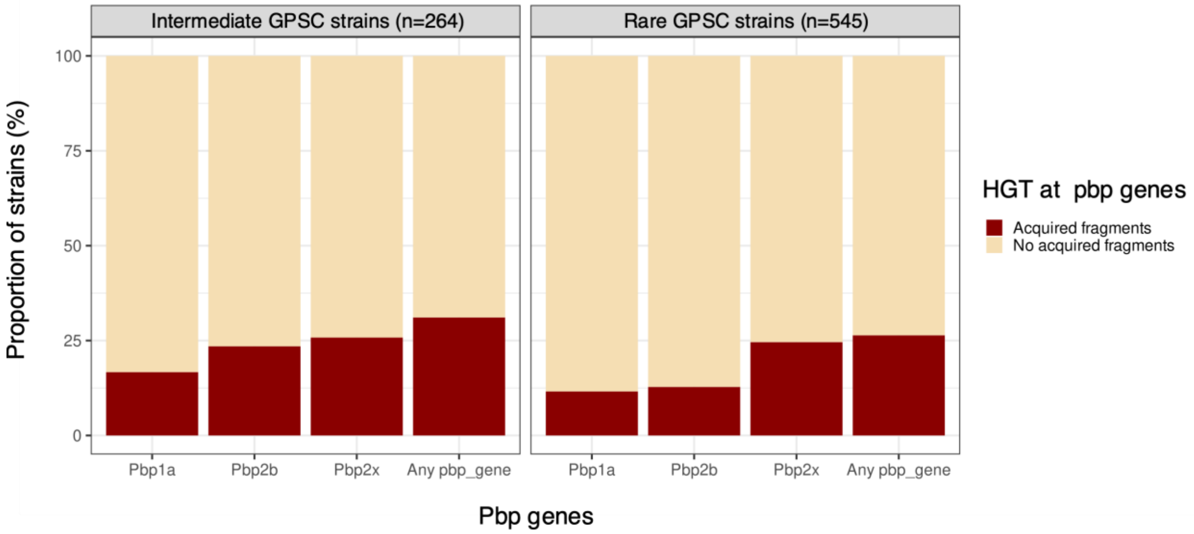

S5 Fig: Proportion of intermediate and rare GPSC strains with acquired fragments in *pbp*1a, *pbp*2b, and *pbp*2x.

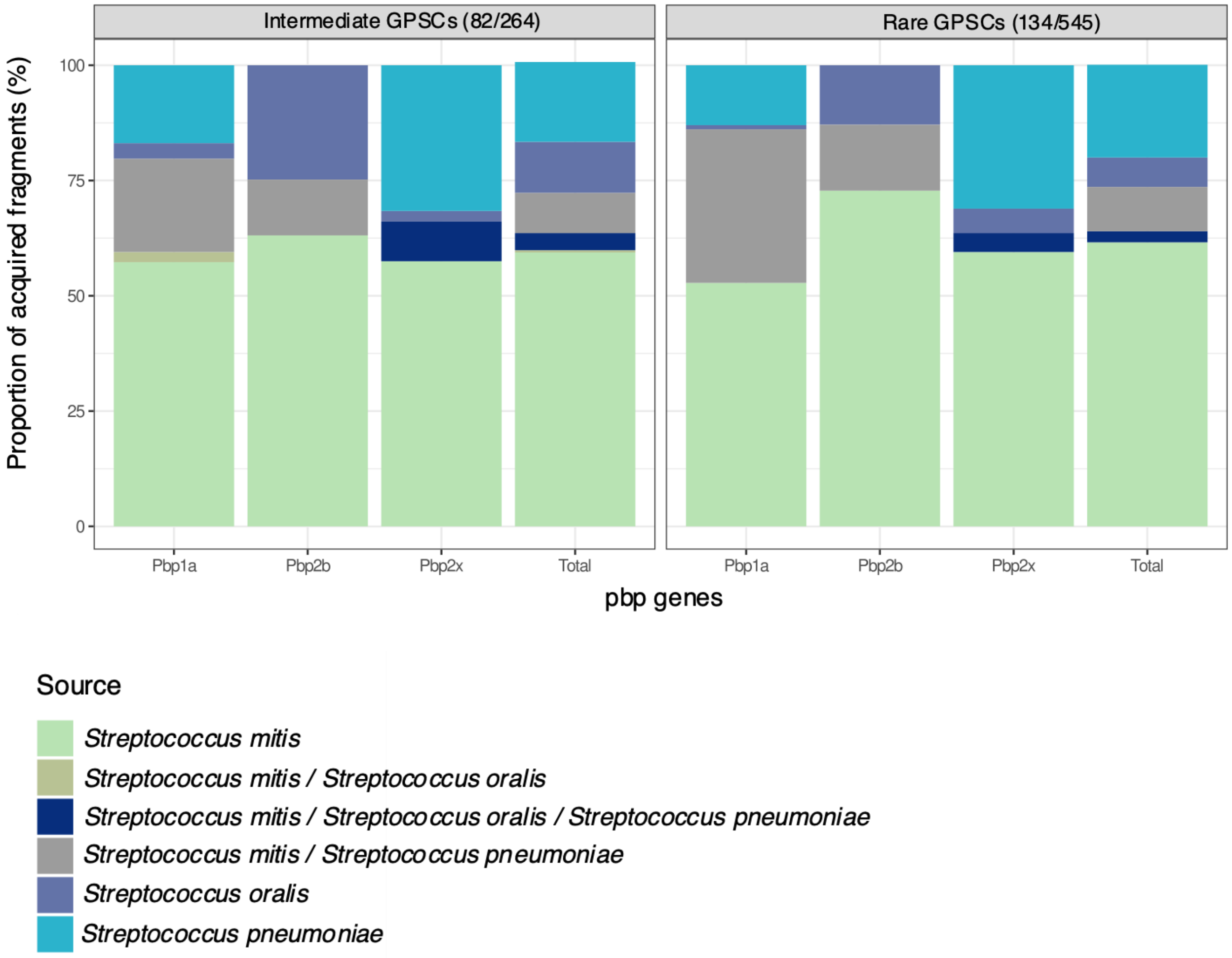

S6 Fig: Donor source proportions of horizontally acquired *pbp* fragments among intermediate and rare GPSC pneumococcal strains.

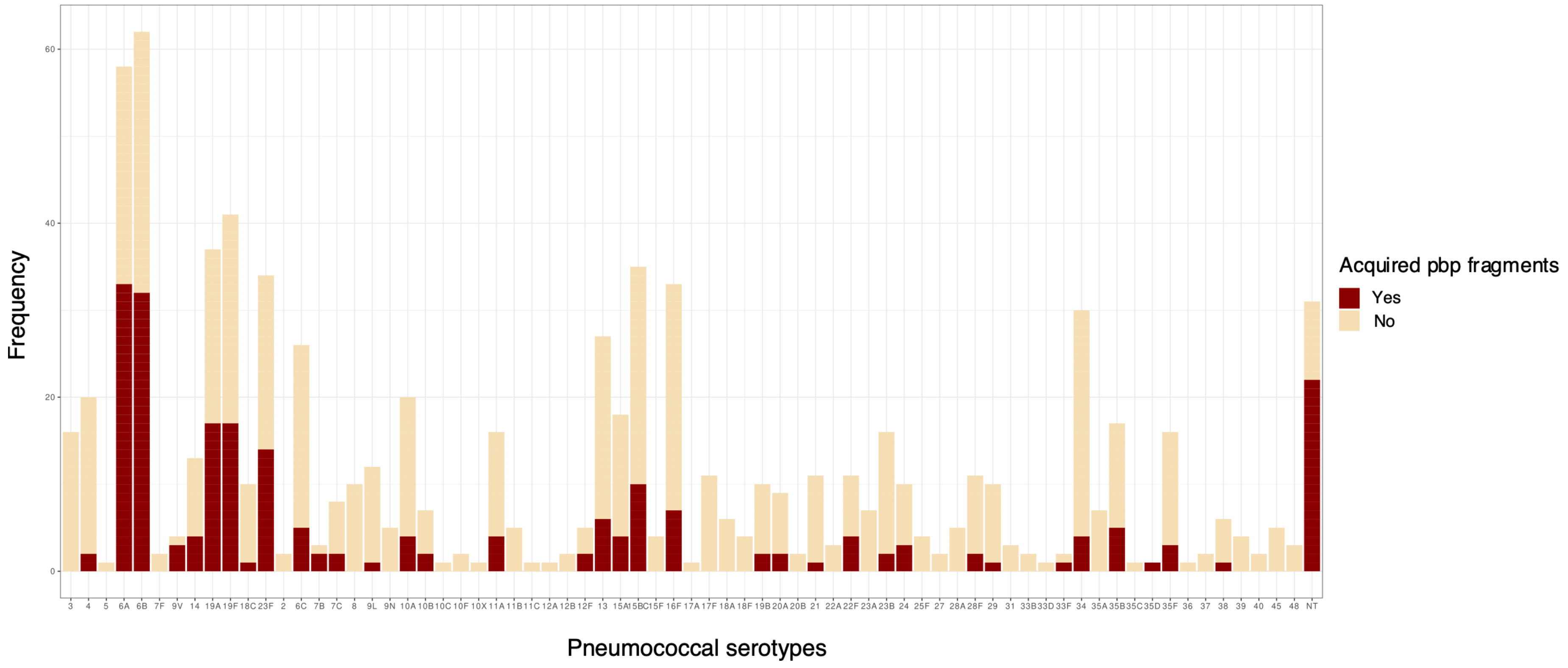

S7 Fig: Frequency of pneumococcal serotypes with evidence of acquired pbp gene fragments.

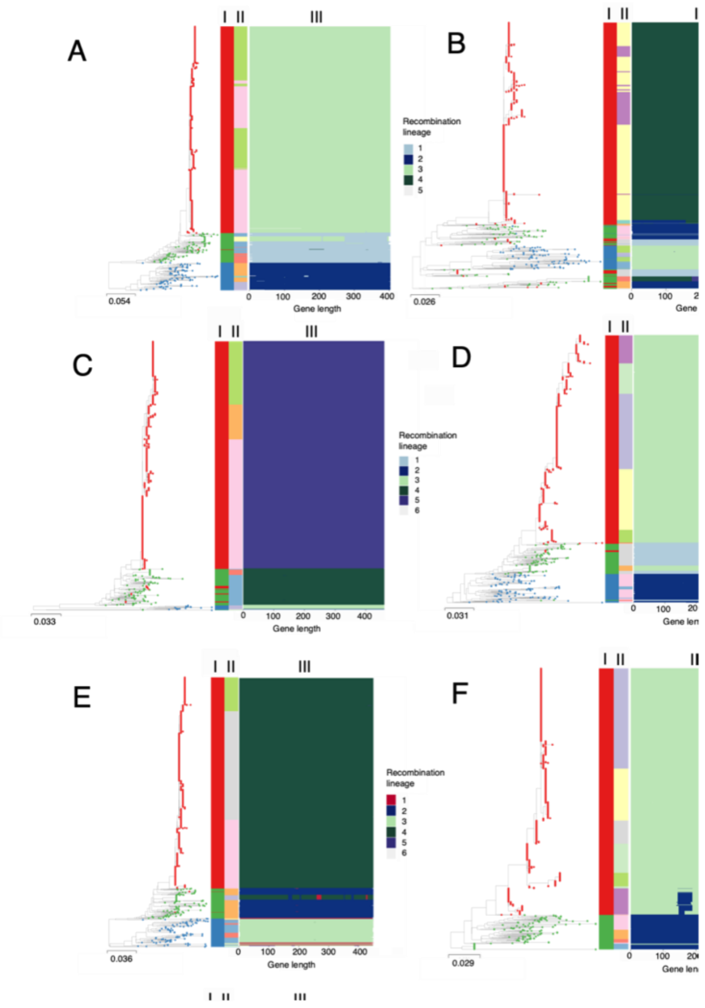

S8 Fig: Horizontal genetic transfer analysis of pneumococcal multi-locus sequence typing (MLST) gene fragments among *S. pneumoniae*, *S. mitis*, and *S. oralis* using the fastGEAR tool. On the left are maximum likelihood phylogenies based on **A**) *aroE* **B**) *ddl* **C**) *gdh* **D)** *gki* **E)** *recP* **F)** *spi* **G)** *xpt* gene alignments of *S. pneumoniae*, *S. mitis*, and *S. oralis.* **I**) Species **II**) Bayesian analysis of population structure (BAPs) clusters of each MLST gene **III**) Recombination (lineage) block panel with HGT fragments identified by FastGEAR over the length of each MLST gene. Blocks of the same colour are hypothesised to be of the same origin.

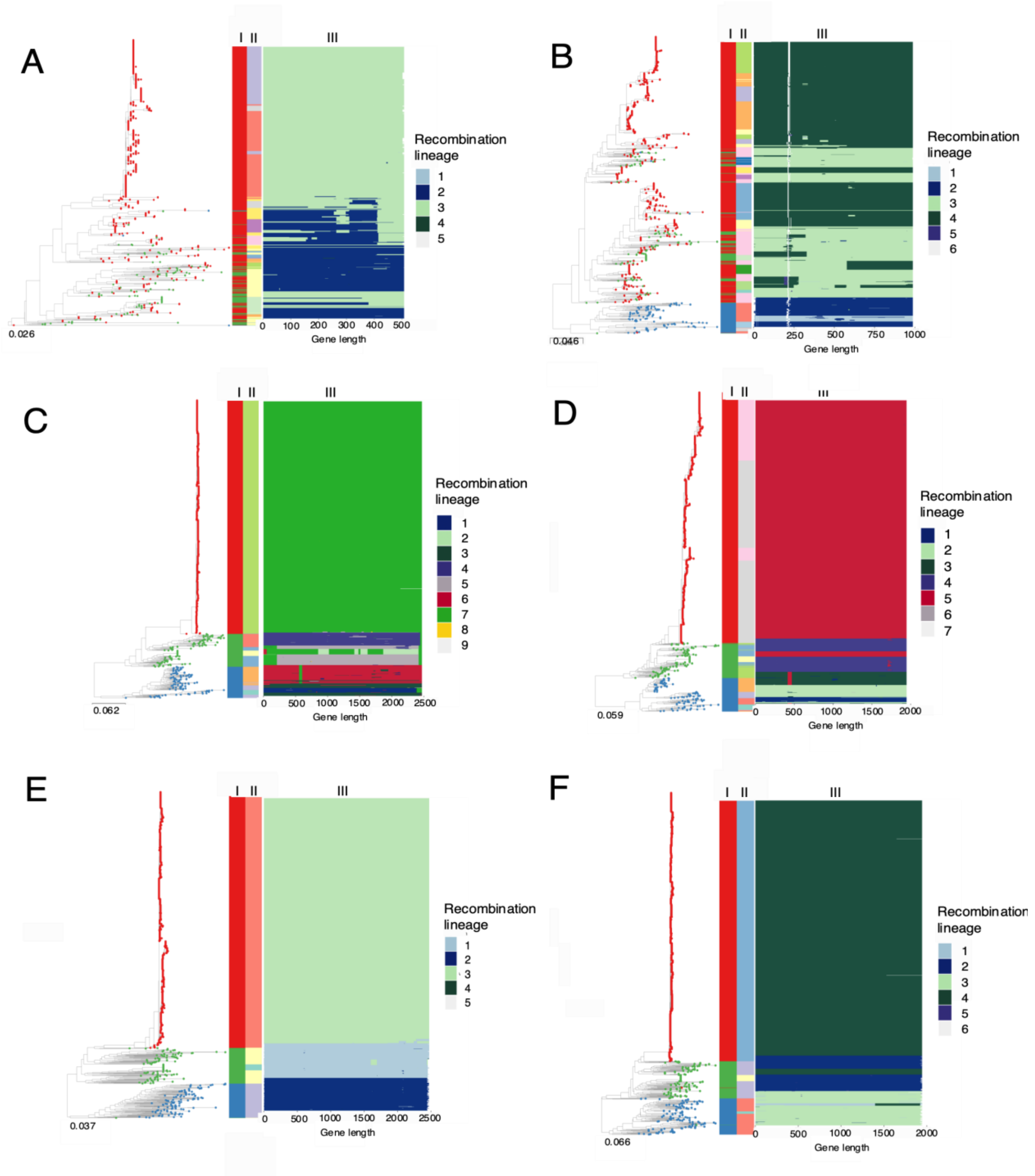

S9 Fig: Horizontal genetic transfer analysis of non-β-lactam antibiotic resistance gene fragments among *S. pneumoniae*, *S. mitis*, and *S. oralis* using the fastGEAR tool. On the left are maximum likelihood phylogenies based on **A**) *folA* **B**) *folP* **C**) *gyrA* **D)** *gyrB* **E)** *parC* **F)** *parE* gene alignments of *S. pneumoniae*, *S. mitis*, and *S. oralis.* **I**) Species **II**) BAPs clusters for each antibiotic resistance gene **III**) Recombination (lineage) block panel with HGT fragments identified by FastGEAR over the length of each antibiotic resistance gene. Blocks of the same colour are hypothesised to be of the same origin.

S1 Table: Number of pneumococcal serotypes, GPSCs, and β-lactam non-susceptible isolates among VTs, NVTs, and non-typeable pneumococci from the 1000 randomly selected Global Pneumococcal Sequencing project isolates.

Vaccine status (PCV13)	Total number of strains	Number of serotypes	Number of GPSCs	Number (and proportion %) of β-lactam non-susceptible strains				
				**PEN**	**AMX**	**CXM**	**CTX**	**CRO**
Vaccine serotypes	439	13	81	161 (36.7)	40 (9.1)	52 (11.8)	36 (8.2)	41 (9.3)
Non-vaccine serotypes	510	48	104	73 (14.3)	17 (3.3)	19 (3.7)	16 (3.1)	16 (3.1)
Non-typeables	51	0	17	30 (58.8)	6 (11.7)	12 (23.5)	3 (5.9)	10 (19.6)

S2 Table: Number and proportion of β-lactam non-susceptible VTs, NVTs, non-typeable pneumococci among the 1000 randomly selected global pneumococcal isolates from the Global Pneumococcal Sequencing project.

Serotype	Total number of strains	Number (and proportion %) of β-lactam non-susceptible strains				
**Vaccine serotypes**		**PEN**	**AMX**	**CXM**	**CTX**	**CRO**
1	45	0 (0)	0 (0)	0 (0)	0 (0)	0 (0)
3	20	4 (20)	0 (0)	0 (0)	0 (0)	0 (0)
4	4	0 (0)	0 (0)	0 (0)	0 (0)	0 (0)
5	5	0 (0)	0 (0)	0 (0)	0 (0)	0 (0)
6A	74	19 (26)	5 (7)	15 (20)	1 (1)	5 (7)
6B	56	16 (26)	2 (4)	3 (5)	1 (2)	2 (4)
7F	5	0 (0)	0 (0)	0 (0)	0 (0)	0 (0)
9V	12	2 (17)	1 (8)	1 (8)	1 (8)	1 (8)
14	34	27 (79)	0 (0)	0 (0)	0 (0)	0 (0)
18C	16	2 (13)	0 (0)	0 (0)	0 (0)	0 (0)
19A	52	28 (54)	2 (4)	3 (6)	3 (6)	3 (6)
19F	52	32 (62)	24 (46)	24 (46)	24 (46)	24 (46)
23F	64	31 (48)	6 (9)	6 (9)	6 (9)	6 (9)
**Non-vaccine serotypes**		**PEN**	**AMX**	**CXM**	**CTX**	**CRO**
6C	37	0 (0)	0 (0)	0 (0)	0 (0)	0 (0)
10A	18	0 (0)	0 (0)	0 (0)	0 (0)	0 (0)
10B	7	0 (0)	0 (0)	0 (0)	0 (0)	0 (0)
11A	39	0 (0)	0 (0)	0 (0)	0 (0)	0 (0)
13	15	9 (60)	0 (0)	0 (0)	0 (0)	0 (0)
15A	34	13 (38)	0 (0)	0 (0)	0 (0)	0 (0)
15B	35	5 (14)	0 (0)	1 (3)	0 (0)	0 (0)
15C	34	3 (8)	0 (0)	0 (0)	0 (0)	0 (0)
16F	29	0 (0)	0 (0)	0 (0)	0 (0)	0 (0)
17F	5	0 (0)	0 (0)	0 (0)	0 (0)	0 (0)
19B	8	1 (13)	0 (0)	0 (0)	0 (0)	0 (0)
20	5	0 (0)	0 (0)	0 (0)	0 (0)	0 (0)
21	18	0 (0)	0 (0)	0 (0)	0 (0)	0 (0)
22A	6	0 (0)	0 (0)	0 (0)	0 (0)	0 (0)
22F	18	0 (0)	0 (0)	0 (0)	0 (0)	0 (0)
23A	22	4 (18)	0 (0)	1 (5)	0 (0)	0 (0)
23B	37	1 (3)	0 (0)	0 (0)	0 (0)	0 (0)
31	5	0 (0)	0 (0)	0 (0)	0 (0)	0 (0)
33F	7	0 (0)	0 (0)	0 (0)	0 (0)	0 (0)
34	27	9 (33)	0 (0)	0 (0)	0 (0)	0 (0)
35B	36	22 (61)	15 (42)	15 (42)	15 (42)	15 (42)
35F	15	0 (0)	0 (0)	0 (0)	0 (0)	0 (0)
38	7	0 (0)	0 (0)	0 (0)	0 (0)	0 (0)
Other*	46	6 (13)	2 (4)	2 (4)	1 (2)	1 (2)
Non-typable	51	30 (59)	6 (12)	12 (24)	3 (6)	10 (20)

S3 Table: Number and proportion of *S. pneumoniae*, *S. mitis*, and *S. oralis* isolates with altered *pbp* binding motifs in reference to conserved *pbp* binding motifs of the β-lactam susceptible *S. pneumoniae* R6.

**Species**	**Altered *pbp* motifs**	**Conserved *pbp* motifs**	**Total number of strains**
S. pneumoniae	301 (30.1%)	699 (69.9%)	1000
S. mitis	120 (85.7%)	20 (14.3%)	140
S. oralis	132 (97.8%)	3 (2.2%)	135

S4 Table: Number and proportion of *S. pneumoniae*, *S. mitis*, and *S. oralis* isolates with altered *pbp*1a*, pbp*2b*, and pbp*2x binding motifs in reference to conserved *pbp* binding motifs of the β-lactam susceptible *S. pneumoniae* R6.

***pbp* gene**	***pbp* motif**	**Species**		
		S. pneumoniae	***S. mitis***	***S. oralis***
*pbp*1a	STMK	94/1000 (9.4%)	9/140 (6.3%)	7/135 (5.2%)
	SRNVP	96/1000 (9.6%)	68/140 (48.9%)	90/135 (66.7%)
	KTG	0/1000 (0%)	0/140 (0%)	0/135 (0%)
*pbp*2b	SVVK	0/1000 (0%)	0/140 (0%)	0/135 (0%)
	SSNT	266/1000 (26.6%)	62/140 (44.3%)	39/135 (28.9%)
	KTGTG	5/1000 (0.5%)	5/140 (3.6%)	5/135 (3.7%)
*pbp*2x	STMK	121/1000 (12.1%)	36/140 (25.7%)	27/135 (20%)
	AHSSNV	93/1000 (9.3%)	45/140 (32.1%)	105/135 (77.8%)
	LKSGT	93/1000 (9.3%)	44/140 (31.4%)	9/135 (6.7%)

S5 Table: Number and proportion of penicillin susceptible and non-susceptible *S. pneumoniae* isolates with altered *pbp*1a, *pbp*2b, *and pbp*2x binding motifs in reference to conserved *pbp* binding motifs of the β-lactam susceptible *S. pneumoniae* R6.

***pbp* gene**	***pbp* motif**	**Susceptible (732)**		**Non-susceptible (264)**	
		**Conserved motifs**	**Altered motifs**	**Conserved motifs**	**Altered motifs**
*pbp*1a	STMK	727	5	175	89
	SRNVP	727	5	175	89
	KTG	732	0	264	0
*pbp*2b	SVVK	732	0	264	0
	SSNT	700	32	32	232
	KTGTG	732	0	259	5
*pbp*2x	STMK	711	21	166	98
	AHSSNV	699	33	204	60
	LKSGT	724	8	181	83

S6 Table: Number and proportion of pneumococcal serotypes with horizontally acquired *pbp* gene fragments.

**Serotype**	**Vaccine status**	**Total number of strains**	**Number of strains with recombined *pbp* gene(s)**	**Proportion (%) of strains with recombined *pbp* gene(s)**
3	VT	20	4	20
5	VT	5	5	100
6A	VT	74	38	51
6B	VT	56	20	36
7F	VT	5	1	20
14	VT	34	30	85
18C	VT	16	4	25
19A	VT	52	30	58
19F	VT	52	35	67
23F	VT	64	39	61
6C	NVT	37	2	5
10A	NVT	18	4	22
13	NVT	15	9	60
15A	NVT	34	14	41
15B	NVT	35	11	31
15C	NVT	34	5	14
16F	NVT	29	14	48
17F	NVT	5	1	20
19B	NVT	8	1	13
20	NVT	5	1	20
21	NVT	18	3	17
22F	NVT	18	1	6
23A	NVT	22	4	18
23B	NVT	36	2	6
34	NVT	27	9	33
35B	NVT	36	23	64
Non-typeable	N/A	51	41	80

S7 Table: Dominant GPSCs and associated pneumococcal serotypes with acquired *pbp*1a, *pbp*2b, and *pbp*2x fragments from *S. pneumoniae*, *S. mitis*, *S. oralis*, or mixed species donors.

Gene	GPSC	Strains in dataset	Strains with recombined *pbp* gene	Dominant serotype	Other dominant serotype	Donor*
*pbp*1a	1	27	1/27	19F (1/1)	-	M, MP
	10	16	16/16	19A (9/16)	3 (4/16)	M, O, MP
	13	23	7/23	6A (7/7)	-	M
	20	33	13/33	23F (13/13)	-	M, O, MP
	21	7	6/7	19F (6/6)	-	M, MP
	59	16	16/16	35B (15/16)	35D (1/16)	M, MP
*pbp*2b	1	27	9/27	19F (8/9)	19A (1/9)	P, M, O
	4	55	12/55	19A (12/12)	-	P
	5	18	17/18	35B (7/17)	23A (4/17)	M, P
	9	39	39/39	14 (25/39)	15A (13/39)	M, P
	20	33	9/33	23F (9/9)	-	P
	45	15	9/15	34 (9/9)	-	M, P
	47	8	7/8	6A (1/7)	6B (6/7)	M
	59	16	16/16	35B (15/16)	35D (1/16)	P
*pbp*2x	1	27	23/27	19F (22/23)	19A (1/23)	M
	4	55	13/55	19A (13/13)	-	M
	5	18	18/18	35B (7/18)	23A (4/18)	M, P
	9	39	37/39	14 (23/37)	15A (13/37)	M
	10	16	16/16	19A (9/16)	3 (4/16)	P
	13	23	10/23	6A (9/10)	6B (1/10)	M, P, MPO
	20	33	22/33	23F (22/22)	-	M, P, MPO
	33	14	14/14	16F (14/14)	-	M
	45	15	9/15	34 (9/9)	-	M, P
	59	16	16/16	35B (15/16)	35D (1/16)	M
	64	20	10/20	6A (10/10)	-	M, P
	65	9	9/9	13 (9/9)	-	M

*Dominant species donors based on fastGEAR *pbp* recombination lineages

M – *S. mitis,* P – *S . pneumoniae,* O – *S. oralis,* MP – *S. mitis* or *S. pneumoniae,* MPO - *S. mitis, S . pneumoniae* or *S. oralis*

S8 Table: Dominant pneumococcal serotypes and associated GPSCs and STs with acquired *pbp* fragments.

Serotype	Dominant GPSCs	STs (frequency)
6A	13, 23, 64	Multiple
13	65	2053 (6/8)
14	9	63* (30/30)
16F	33	9568 (7/8), 11770 (4/5)
19A	4, 10	199 (9/38)
19F	1, 21	4414 (12), 236 (5/5)
23F	20	802 (9/16), 4413 (7/7)
34	45	1439 (7/9)
35B	5, 59	558 (12/13)

*PMEN25 (ST 63) – Malawi (7/30), Gambia (6/30), Thailand (5/30), UK (3/30), USA (9/30)

S9 Table: The coverage of intermediate and rare GPSCs in the supplementary analysis in comparison to the larger GPS project dataset

GPSC type	Number of GPSCs per GPS type	Number of strains in GPS dataset per GPSC type	Epidemiological sampling approach		Phylogenetic sampling approach	
			GPSCs represented	Number of strains	GPSCs represented	Number of strains
Dominant	35	8356/13454	34/35 (97%)	480/1000	-	-
Intermediate	132	4149/13454	67/132 (51%)	355/1000	132/132 (100%)	264/809
Rare	371	950/13454	49/371 (13%)	165/1000	371/371 (100%)	545/809

S10 Table: Number and proportion of *S. pneumoniae*, *S. mitis*, and *S. oralis* isolates with horizontally acquired gene fragments among pneumococcal MLST genes (*aroE*, *ddl*, *gdh*, *gki*, *recP*, *spi*, *xpt*) and non-*pbp* antibiotic resistance genes (*folA*, *folP*, *gyrA*, *gyrB*, *parC*, *parE*) identified by the fastGEAR tool.

**Gene**	**Number (and proportion %) of strains with recombined genes for each species**		
	S. pneumoniae	***S. mitis***	***S. oralis***
aroE	10/1000 (1.0)	27/140 (19.3)	13/134 (9.7)
ddl	17/1000 (1.7)	26/139 (18.7)	3/114 (2.6)
gdh	0/1000 (0)	2/139 (1.4)	0/19 (0)
gki	0/1000 (0)	4/140 (2.9)	2/132 (1.5)
recP	2/1000 (0.2)	28/140 (20)	4/134 (3.0)
spi	90/1000 (9)	23/140 (16.4)	0/0 (0)
xpt	10/998 (1.0)	9/139 (6.5)	27/134 (20.1)
folA	198/1000 (19.8)	23/140 (16.4)	2/4 (50)
folP	280/998 (28.1)	72/140 (51.4)	28/133 (21.1)
gyrA	5/997 (0.5)	40/140 (28.6)	62/133 (46.6)
gyrB	2/996 (0.2)	24/138 (17.4)	42/133 (31.6)
parC	19/998 (1.9)	44/140 (31.4)	11/133 (8.3)
pare	0/997 (0)	7/138 (5.1)	24/133 (18.0)
